# Use of Cluster Analysis for Identifying Metafounders

**DOI:** 10.1111/jbg.70039

**Published:** 2026-01-23

**Authors:** Christine Anglhuber, Christian Edel, Eduardo C. G. Pimentel, Reiner Emmerling, Kay‐Uwe Götz, Georg Thaller

**Affiliations:** ^1^ Bavarian State Research Center for Agriculture Institute for Animal Breeding Grub Germany; ^2^ Christian‐Albrechts‐Universität Institute for Animal Breeding and Husbandry Kiel Germany

**Keywords:** genetic evaluation, metafounder, single‐step genomic BLUP

## Abstract

In the metafounder approach, the relationship matrix of metafounders, Γ is used to transfer information on relationships between pedigree founders into the numerator relationship matrix A, creating matrix $A^{\Gamma }$. Commonly metafounders are defined based on the available information of the animal (e.g., country, sex, breed) similar to unknown parent groups (UPG). This limits the ability of metafounders to correctly reflect the population structure. In Single‐Step Models, hidden stratification in the population may cause inconsistencies between matrix A and the genomic relationship matrix G when they are combined into matrix H. Reliable information on the true structure in a population can be obtained from genotypes. In this study, we investigate an approach to transfer information on population structure from the genotyped animals to the ungenotyped ancestors. We used an unsupervised clustering approach to assign pedigree founders to metafounders and performed Single‐Step genomic evaluation for an increasing number of metafounders (nMF) assumed. The optimum nMF to model was determined by harmonising the trend in inbreeding in G and $A^{\Gamma }$ and by monitoring of elements in Γ. A semi‐stochastic simulation based on real genotypes from Fleckvieh was used to investigate two scenarios: a trait with a strong genetic trend and a trait with no genetic trend. The quality of the prediction was determined by a regression of true breeding value as obtained from the simulation on estimated breeding value. The modelling of metafounders defined by population structure analysis led to a slight reduction in prediction quality in a trait with no trend, but was still stable in the range of the optimum nMF. In a trait with a strong genetic trend, prediction qualtity was improved compared to a common Single‐Step model. The largest improvement was achieved in the range of the proposed optimum nMF.

## 1 Introduction

In Single‐Step GBLUP, the numerator‐relationship‐matrix A and the genomic relationship matrix G are combined into matrix H (Legarra et al. 2009). The combined relationship‐matrix H can be divided into two components, where the first one is a covariance matrix calculated based on observed and imputed gene‐contents for animals in the pedigree and the second one is a covariance structure modelling an additional random effect termed the ‘imputation‐residual’ (Fernando et al. 2014; Christensen and Lund 2010). In this context ‘imputation of gene‐contents’ is used to describe a simple regression conditional on observed gene‐contents using numerator relationship matrix A (Gengler et al. 2007). Matrix H therefore establishes a rudimentary form of the true relationship of pedigree founders as a combination of the covariance of imputed gene‐contents and the covariance of the imputation residuals.

Ideally, the earliest ancestors in A (i.e., the pedigree founders) should belong to an unselected and unrelated base population. The evolution of the Mendelian‐Sampling variance via the increase in inbreeding and relatedness is then described conditional on this initial assumption. However, in practice the base of the pedigree depends on the availability of pedigree data and cannot fulfil all the required assumptions. Furthermore, missing pedigree information throughout generations is a common problem in dairy populations. To avoid bias in estimates, animals with missing ancestry are assigned to unknown parent groups (UPG) to account for different genetic levels not represented in A (Quaas and Pollak 1981). Historically, these UPG were commonly defined based on the available information, such as breed, country, sex and birth year of the animal. For the definition of UPG the number of animals per group is important, to assure estimability of group effects. This limits the number of UPG that can be modelled. Such a rough grouping of animals leads to loss of information on the true structure of these animals and UPG only reflect a share of this true structure.

Setting up a matrix G from SNP‐marker‐genotypes like in VanRaden (2008) requires a form of centring and scaling of individual gene‐contents. Depending on the way this centring and scaling is performed, coefficients in G may or may not relate to the observable heterozygosity of the animals at genomic markers. Assuming a uniform base allele frequency of 0.5 for all markers, the resulting genomic inbreeding coefficients directly relate to individual average heterozygosities at genomic markers (for details see Anglhuber et al. 2024). These individual inbreeding coefficients can be averaged over cohorts to describe the change in the average level of heterozygosity over generations. Off‐diagonals of such a G consistently describe the co‐segregation between animals even in the presence of population stratification (Anglhuber et al. 2024; Christensen 2012; Legarra et al. 2015; Forni et al. 2011). Although not easily accessible, this matrix therefore also contains information on mild or strong forms of population‐subdivision, with respect to the genotyped animals per se but also, indirectly, with respect to the pedigree founders that in most cases are not genotyped. For a polygenic trait it is reasonable to assume that this information, if recovered from the marker‐genotypes, also provides a valid description of corresponding conditions and processes at the unknown QTL‐genotypes linked to them.

This information which is implicitly part of G is missing from A as is all information on relationships not accounted for in the recorded pedigree. Several strategies have been proposed to account for systematic differences between G and A to facilitate the construction of H (Legarra et al. 2015; Christensen 2012; Forni et al. 2011; Vitezica et al. 2011). Metafounders can be described as discernible gene‐pools or lines from which individual pedigree founders have descended. The metafounder approach (Legarra et al. 2015) also provides the methodological framework to characterise and describe these gene‐pools or lines and to trace this information across the pedigree to construct an enhanced version of the numerator‐relationship matrix. This is accomplished by using an estimated matrix of self‐relationships of metafounders (Γ) as a kernel, assigning pedigree founders to metafounders and then proceeding with standard procedures based on pedigree data. The resulting $A^{\Gamma }$ now accounts for the initial genetic structure already present in the pedigree base. This approach provides the prerequisites required to harmonise conventional and genomic models in a consistent way. It is particularly useful with respect to the evaluation of genotyped and non‐genotyped animals in a Single‐Step model using the combined relationship matrix H.

However, the process of identification or definition of metafounders is not straightforward. Many studies define metafounders the very same way as the UPG currently applied in their classical evaluations and simply model them in another way by using them to construct $A^{\Gamma }$ (Kudinov et al. 2020, 2022; Bermann et al. 2021; Bradford et al. 2019). This strategy limits the number of metafounders to be modelled by the number of UPG, which has to be restricted due to estimability concerns. For instance, a possible scenario would be that all ancestors up to the base are known and each ‘missing’ founder would be represented by himself. In this sense the number of pedigree founders is the upper boundary to describe the structure (true relationships) among them. The metafounder approach gives the freedom to model as many metafounders as necessary to describe this structure. Most importantly, defining metafounders the same way as UPG restricts the metafounder approach to further hold to arbitrary prior believes based on sex, country and birth year, which have been used in conventional models because no other information was available. These approaches ignore the information contained in the genotypes, which is the most reliable source for identifying stratification within a population. In a recent publication we proposed the definition of metafounders based on genomic population structure analysis (Anglhuber et al. 2024). Our approach improved the compatibility of $A_{22}$, the submatrix of genotyped animals, and matrix G in European Brown Swiss. However, in this initial study, population structure analysis was only performed for genotyped animals, without providing a method for how stratification information can be transferred from genotyped animals to pedigree founders and finally to metafounders. The assignment of pedigree founders to metafounders is a prerequisite to make full use of all features of the metafounder approach under practical conditions (Legarra et al. 2015). In practice, only recent generations of individuals are routinely genotyped. Pedigree founders and their immediate offspring are often not genotyped, requiring the development of methods to extrapolate the information from the genotyped animals to their non‐genotyped ancestors.

In this investigation we propose a metafounder approach that differs from the concept of UPG. Specifically, we aimed at answering the following questions regarding our concept of metafounders: 1. Is it possible to identify metafounders solely relying on a cluster algorithm? 2. If yes, what are possible criteria for defining an optimum number of metafounders? 3. To what extent can prediction quality be improved through the integration of such metafounders in a Single‐Step model?

We use a semi‐stochastic simulation to demonstrate the use of metafounders in an information‐dense scenario and to evaluate the approach when the number of metafounders reaches the number of pedigree founders. Instead of arbitrary criteria such as birth year and origin, metafounders are defined according to information extracted from genomic data. We provide a method for transferring information from the genotyped animals to the ungenotyped pedigree founders and present strategies to independently determine an optimum number of metafounders based on properties of G and $A^{\Gamma }$. We further elaborate why and in which cases it is advantageous to use metafounders in Single‐Step models and highlight several aspects and consequences relevant to the application of the metafounder approach in practice. Finally, we determine the feasibility of the approach when limits of estimability were reached and assess the effect on EBV.

## 2 Materials and Methods

The genotypes used in this investigation were collected routinely as part of breeding value estimation; a separate approval by an Institutional Animal Care and Use Committee or Institutional Review Board was not necessary.

All steps of the analysis were performed in R (v. 3.5.2) (R Core Team 2018) and graphs were made with ggplot2 (Wickham 2016).

### 2.1 Selection of Study Population

**FIGURE 1:**
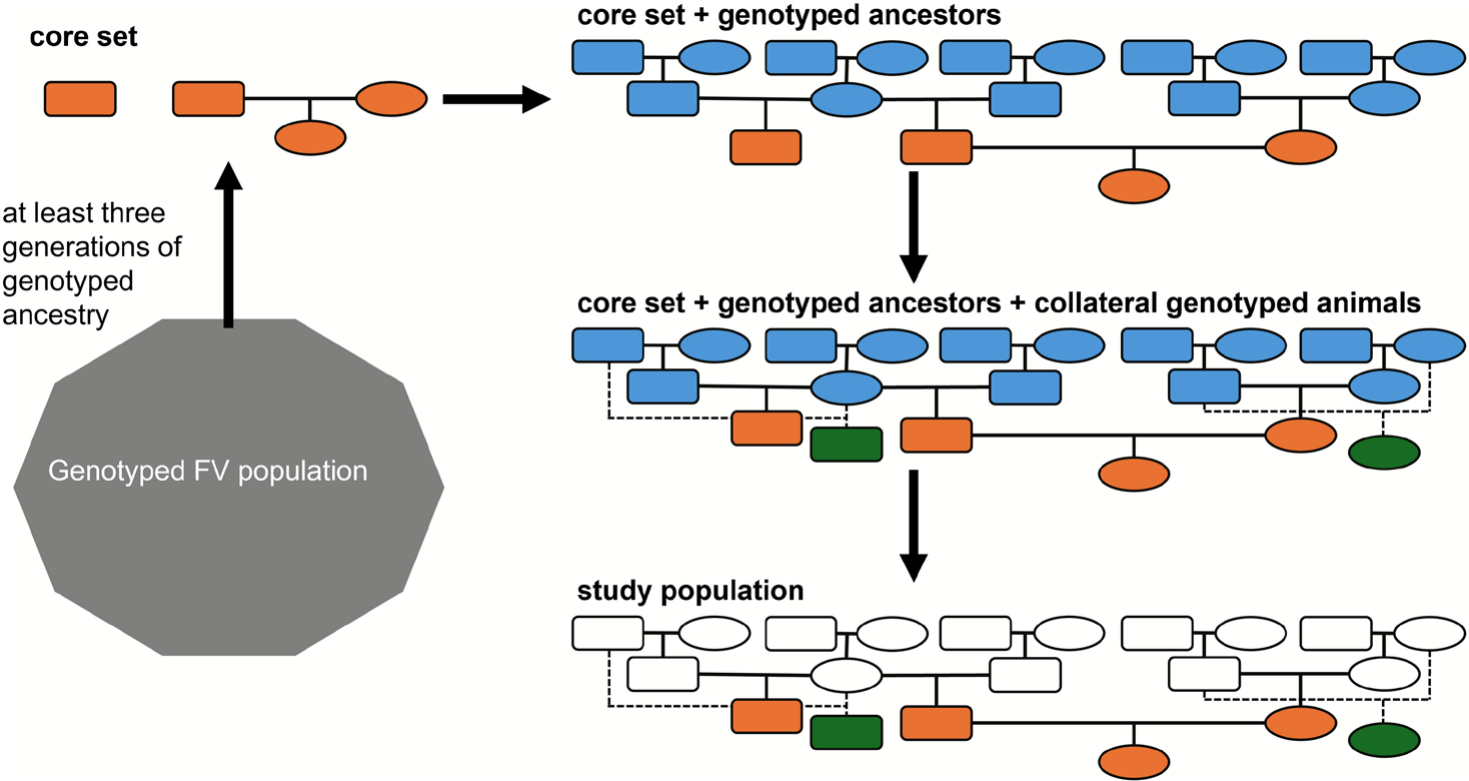
Illustration of the steps to select the study population. Rectangles represent males, ellipses represent female individuals. Colour represents different groups of animals: Orange for the core set, blue for the genotyped ancestors of the core set, green for the genotyped collateral animals. In the final set, only animals retaining their genotype for the optimisation pipeline are coloured. [Colour figure can be viewed at wileyonlinelibrary.com]

This study was performed as a semi‐stochastic simulation based on real Fleckvieh genotypes and pedigrees. Figure 1 provides an illustration of the steps included in the selection of the study population. From the 721,021 genotypes currently used in the joint German‐Austrian‐Czech genomic evaluation (April 2024), animals with at least three generations of fully genotyped ancestors were selected as a core set containing 3370 individuals. Then we also included these fully genotyped ancestors, which were in total 5370. In the last step 19,066 genotyped collateral animals were added to obtain the final set. Animals at the base of this pedigree were defined as pedigree founders (PF, $n_{\mathrm{PF}}$ = 1505). This resulted in a fully genotyped pedigree of 27,806 related animals (n) spanning over several generations. True breeding values were simulated for all these animals. In all other steps of the analysis, all genotypes of PF and direct offspring were masked. More details are explained below.

In routine genotyping of Fleckvieh animals, an Illumina BovineSNP50 BeadChip (Illumina, San Diego, CA) is used. Routine SNP quality control included removing SNPs with call rates below 90%, minor allele frequencies below 2%, or which deviated from Hardy–Weinberg equilibrium, resulting in 40,859 SNPs in analysis.

### 2.2 Simulation of True Breeding Values

To simulate the true breeding value (TBV) for a trait with a strong genetic trend (scenario ‘trend’) we assumed that the trait was determined by 1000 QTL with additive gene‐effects only. A subset of 1000 SNP‐markers were chosen to represent these QTL. In order to select a SNP‐marker as a QTL we required it to show a consistent change in allele‐frequency over time. This was evaluated by a regression of mean allele frequencies on birth years (2012 to 2024). A consistent trend was determined by a fit of the regression larger than 0.3 (see Appendix A). The 1000 QTL‐SNPs were sampled from all SNPs with a clear trend (22,964). Gene‐effects were sampled from $N\left(0,\left(2*\sigma_{a}^{2}/1000\right)\;\right)$, with $\sigma_{a}^{2}$ = 1. Although the selection of QTL‐SNPs was performed only once, the sampling of gene effects was repeated for 500 times. In each repetition, the sign of the gene effect was aligned according to the change in allele frequency to produce a positive trend in the trait mean. This approach guarantees a positive genetic trend in line with allele‐frequency changes. However, the magnitude of the trend cannot be predetermined. Some of this observed change in allele frequencies might be due to drift, though this is hard to quantify.

TBVs for each animal were then calculated as the sum of the product of each gene effect and the genotype of each animal using 0, 1, 2 coding (VanRaden 2008) resulting in 500 repetitions of TBV for each animal. In each repetition, the mean TBV of all PF was set to 0 and rescaled to ensure an additive genetic variance of 1 among the PF. Phenotypes were then calculated as TBV plus a random factor drawn from $N\left(0,\sigma_{e}^{2}\right)$, with $\sigma_{e}^{2}$ set to fulfil: $\frac{\sigma_{a}^{2}}{\sigma_{a}^{2}+\sigma_{e}^{2}}=h^{2}=0.25$ in PF.

Since in practice some traits do not show genetic trend, we additionally investigated a scenario without trend (scenario ‘notrend’). For this scenario, a random set of 1000 SNPs was selected from all available SNPs. Gene effects were sampled in the same manner as described above, but without manipulating the sign of the sampled gene effects.

In all further steps of the investigation, the QTL‐SNPs used to simulate TBV were masked in either scenario.

### 2.3 Study Design

After generating TBV and phenotypes and with genotypes available for all animals in the design, we defined different groups of animals with different levels of information to mimic ‘realistic’ conditions. Genotypes of PF were masked. For direct offspring of PF, genotypes were also masked to introduce a realistic level of uncertainty in the estimation of gene contents of PF. All other animals kept their observed genotype (GT). From here on we will use the labels ‘genotyped’ and ‘non‐genotyped’ to address these groups. Females and all male animals with offspring were assigned to be phenotyped (PT), with the phenotype simulated as a single observation of own performance, as described above. This resulted for example in all PF and their direct offspring providing phenotypes but no genotypic information, while the most recent generation of male animals provided genotypes only. As above, we will use the labels ‘phenotyped’ and ‘non‐phenotyped’ to address the resulting groups in the following. Overall, 22,316 animals were labelled genotyped, 16,980 as phenotyped and 11,490 animals as being genotyped and phenotyped (Table 1).

**TABLE 1:** Overview data structure.

Type	Class	Genotyped	Phenotyped	Group	*N*
Conceptual	Metafounder	No	No		nMF
Real	PF	No^a^	Yes	PTnoGT	1505
Real	Progeny of PF	No^a^	Yes	PTnoGT	3985
Real	Parents	Yes	Yes	PTandGT	11,490
Real	Candidates	Yes	No	GTnoPT	10,826

^a^ Genotypes available but masked.

### 2.4 Imputing the Gene Content of Pedigree Founders

Due to the study design, genotypes of PF were masked. In the proposed approach we defined metafounders solely based on genomic information and assigned PF to metafounders. For this purpose, the available genomic information of genotyped animals needs to be transferred to the non‐genotyped PF in some way. Imputation of gene content of non‐genotyped animals can be done based on observed genotypes and the available pedigree (Gengler et al. 2007). This is an underlying function in the construction of H (Fernando et al. 2014). Following their notation:

(1) $$M_{1}=A_{12}A_{22}^{-1}M_{2}$$

with subscripts 1 and 2 referring to non‐genotyped and genotyped individuals, respectively and matrix $M_{2}$, the centred matrix of genotypes. For centring we assumed p=0.5. Since $M_{1}$ contained PF and their direct offspring, only the submatrix relating to PF was kept for analysis. Including such an ‘imputation’ step in the analysis reflects the situation for many breeding populations, where pedigree founders are not genotyped. The imputed genotypes were only used to inform the clustering algorithm described in the following section and were not used in any other steps of the investigation.

### 2.5 Definition of Metafounders and Construction of Γ

K‐means clustering is a popular choice of approach in exploratory data analysis. It identifies cluster centres and assigns subjects to clusters to maximise the similarity within the cluster and minimise similarity between clusters while simultaneously updating the cluster centres until convergence is reached (Morissette and Chartier 2013). Function kmeans() implemented in R (R Core Team 2018; Hartigan and Wong 1979) provides as results the assignment of subjects to clusters, the centre means and sum of squares (sums of within, between and total difference for individual to cluster centre) for each cluster. We found that the choice of the appropriate cluster algorithm is a critical aspect with respect to the validity of the obtained clustering and determines its usefulness for the definition of metafounders. In preliminary tests, we identified the Hartigan–Wong algorithm with the number of initial samples for centre estimation set to 10 and limiting the maximum number of iterations performed to 10*number of subjects to be a good compromise with respect to consistency and repeatability of the resulting cluster assignments and the computing time needed. The function relies on a predefined number of assumed clusters or a matrix of cluster centres for each of the discriminatory variables used (Hartigan and Wong 1979).

The cluster analyses were performed using the imputed gene‐contents of PF as discriminatory variables. In each step of the analysis, an assumed number of clusters (nMF = number of assumed metafounders) was defined, and PF were assigned to one of the nMF clusters. On the basis of this assignment, all further steps of investigation described below were performed. After that, nMF was increased and, after re‐clustering the PF, all subsequent steps were repeated. To ensure reproducibility, all clustering was performed with a seed set specific to each nMF.

Based on the assignment of PF to a metafounder cluster, a matrix Q (n × nMF ), defining the contribution of each metafounder to each (genotyped) animal was set up to estimate metafounder allele frequencies using a GLS approach (Gengler et al. 2007; Aldridge et al. 2019; Plieschke et al. 2016). Using these estimates matrix Γ was constructed relating to a base population of maximum heterozygosity under Hardy–Weinberg equilibrium (Garcia‐Baccino et al. 2017; Legarra et al. 2024b). Finally, $A^{\Gamma }$ was calculated as described in (Legarra et al. 2015) and used to set up a modified $H^{\Gamma }$ to estimate Single‐Step breeding values.

### 2.6 Setting Up Single‐Step Equations

#### 2.6.1 Model

For each nMF, estimation of breeding values (EBVs) was performed with a Single‐Step GBLUP model and the following mixed model equations:

$$\left[\begin{array}{cc} 1^{\prime }1 & 1^{\prime }Z\\ Z^{\prime }1 & Z^{\prime }Z+{\left(H^{\Gamma }\right)}^{-1}\lambda \end{array}\right]\left[\begin{array}{c} \mu \\ \hat{a} \end{array}\right]=\left[\begin{array}{c} 1^{\prime }y\\ Z^{\prime }y \end{array}\right]$$

where y is a vector of phenotypes, μ is the fixed effect, Z is the design matrix relating observations to breeding values, $\lambda =\left(\frac{1-h_{\text{resc}}^{2}}{h_{\text{resc}}^{2}}\right)$ and $\hat{a}$ is the vector of breeding values. The rescaled heritability used, $h_{\text{resc}}^{2}$ is the heritability relating to the appropriate $\sigma_{a_{\text{resc}}}^{2}$ for each nMF. The rescaling of $\sigma_{a}^{2}$ is described in the next section. The inverse of the combined relationship matrix $H^{\Gamma }$ was constructed with $A^{\Gamma }$ as:

$${\left(H^{\Gamma }\right)}^{-1}={\left(A^{\Gamma }\right)}^{-1}+\left[\begin{array}{cc} 0 & 0\\ 0 & G^{-1}-{\left(A_{22}^{\Gamma }\right)}^{-1} \end{array}\right]$$

Matrix G was set up following VanRaden's approach 1 (2008) with allele frequencies set to 0.5 and number of markers, m = 39,859. To provide a baseline, the results for a simple conventional animal model based on pedigree data only (with covariance given by A, and nMF = 0) were included in the tables. Modelling a single metafounder (covariance structure is $H^{\Gamma }$ and nMF = 1) is equivalent to the common approach in Single‐Step applications. This is because at least one metafounder is required to make a matrix A (referring to average heterozygosity of PF) compatible to a genomic relationship matrix using a uniform base‐allele frequency of 0.5, which implies a purely conceptual base population of maximum heterozygosity under Hardy–Weinberg conditions (Anglhuber et al. 2024). Only with nMF ≥ 2 additional structural information is transferred from genotyped animals to PF.

#### 2.6.2 Rescaling the Additive‐Genetic Variance

In a Single‐Step model, the combination of G and A requires that both matrices refer to the same genetic base and the same value for the additive genetic variance (Christensen 2012; Legarra et al. 2015). Within the metafounder approach, it is convenient to rescale matrix A that refers to a specific set of founder animals to G. When G is constructed assuming a uniform base allele frequency of 0.5, it refers to an idealised base with maximum average heterozygosity under Hardy–Weinberg conditions. The process of rescaling of A requires an estimate or an assumption about the average heterozygosity of the specific set of pedigree founders and is achieved by establishing a single metafounder (γ) (Anglhuber et al. 2024; Legarra et al. 2015). The additive genetic variance that was originally estimated referring to this same set of founder animals must then be rescaled accordingly.

Introducing more than one metafounder implies relaxing the assumption of an unstructured or unrelated pedigree base underlying the conventional model. For instance, if there are two populations that differ in their means, the variance of animals produced from the cross (even after many generations) will be different from the variance in each population. As a consequence, structural information and its contribution to the overall genetic variance becomes part of A, operationalised by the formulation and use of $A^{\Gamma }$. The additive genetic variance that was originally estimated under the wrong assumption of an unstructured pedigree base must be rescaled since the variance of group or strata means has contributed to the estimate. More explicitly, the additive‐genetic variance in the model must be corrected for this contribution to avoid double‐counting since $A^{\Gamma }$ already reflects the structural variation associated with it.

The rescaling of the additive‐genetic variance used here is based on elements of Γ and was originally derived by Legarra et al. (2015):

σaresc2=σa21−τ2

with $\tau =-2*\left(\frac{\bar{\mathrm{diag}\left(\Gamma \right)}}{2}-\bar{\Gamma }\right)$, resulting in σa21−γ2fornMF=1σa21+diagΓ¯2−Γ¯fornMF>1 (Legarra et al. 2015).

The parameter τ provides a measure of the ratio of diagonal and off‐diagonal values in the respective Γ matrices.

For the rescaling of $h^{2}$, $\sigma_{e}^{2}$ which was sampled for the generation of TBV was used $h_{\text{resc}}^{2}=\frac{\sigma_{a_{\text{resc}}}^{2}}{\sigma_{a_{\text{resc}}}^{2}+\sigma_{e}^{2}}$.

### 2.7 Assessment of Results

**FIGURE 2:**
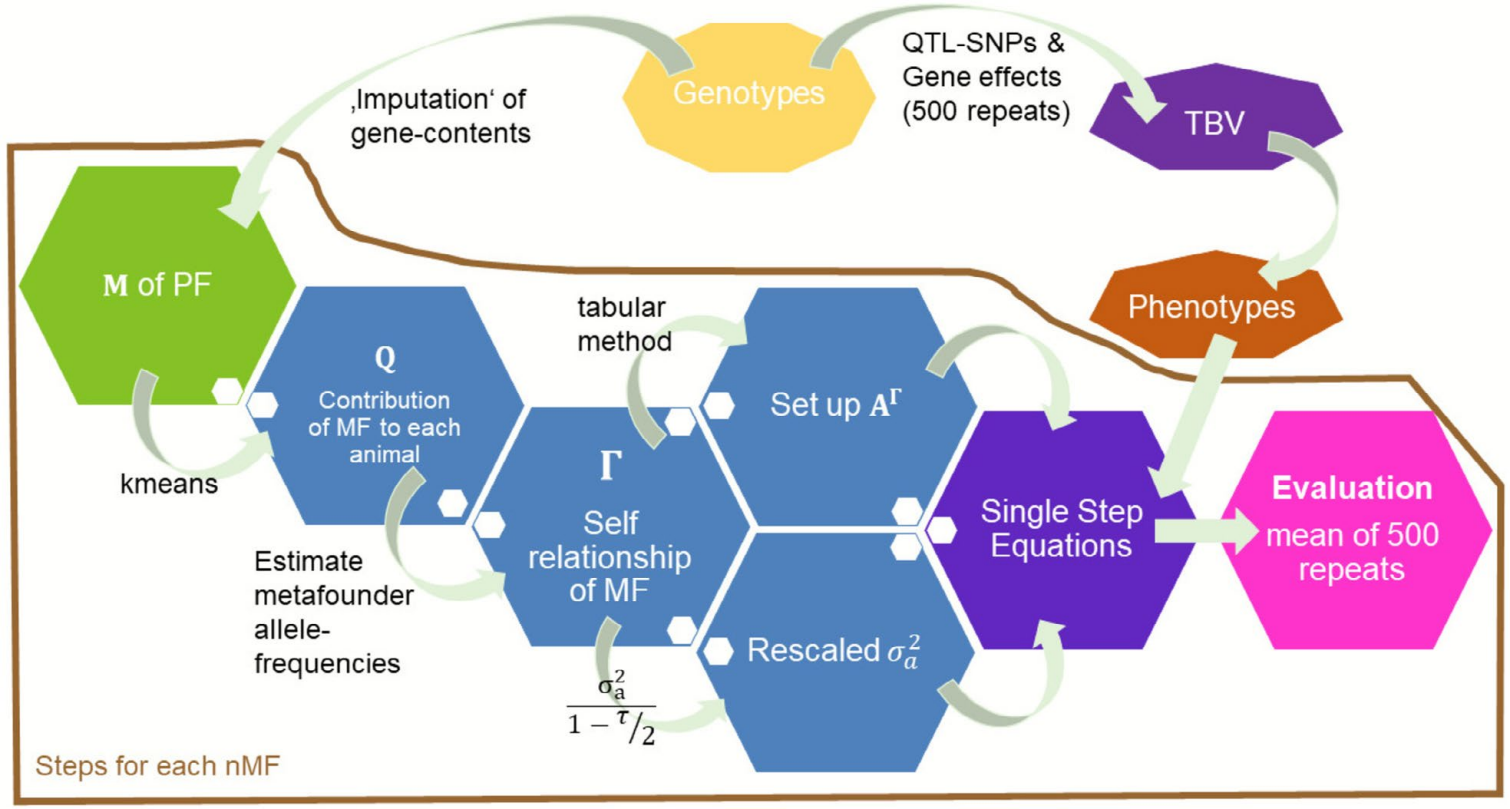
Depiction of the simulation steps (outside of brown box) and the optimisation process for each selected number of metafounders (inside of brown box). [Colour figure can be viewed at wileyonlinelibrary.com]

Evaluation of the results from our simulation runs was based on the regression of TBV on EBV. We analysed the intercept (a), the slope of the regression (b), the fit of the regression (*R* ^2^) and the bias, calculated as mean of EBV minus TBV within each repetition. Expected optimum values for a and bias are 0, and 1 for the slope of regression. Estimates of b>1 indicate an underdispersion of breeding‐values, whereas values of b<1 indicate overdispersion, sometimes also referred to as ‘trend bias’ (Mäntysaari et al. 2010). Results presented here are means of 500 repetitions of sampling of gene effects, i.e., each animal obtained 500 TBV, 500 phenotypes and 500 GEBVs. We present results for four groups of interest: all animals (all), animals with PT but without GT (PTnoGT); animals with GT but without PT (GTnoPT); animals with GT and PT (PTandGT). A schematic summary of the implemented pipeline is given in Figure 2. Correlations of EBV for nMF = 1 and all other nMF were computed to assess the effect of including metafounders on the ranking of animals.

For the sake of a more compact visual presentation, *R* ^2^ in Figure 8 is displayed as marginal *R* ^2^ ($R_{m}^{2}$) where:

$$R_{m}^{2}=\frac{R_{i}^{2}-R_{b}^{2}}{R_{i}^{2}*R_{b}^{2}+1-2*R_{b}^{2}}$$

with $R_{i}^{2}$ as the specific *R* ^2^ for each scenario, group and nMF, and $R_{b}^{2}$ is the *R* ^2^ for each scenario and group for nMF = 1. This allows to become independent of the base line level of *R* ^2^ (Edel et al. 2016), which differs for different groups and also between scenarios ‘trend’ and ‘notrend’. Unscaled values of *R* ^2^ for all groups and scenarios are available from Table 6.

### 2.8 Derivation of a Stopping Criterion

To make practical use of an unsupervised analysis, a stopping criterion for the optimisation process is required. Kmeans provides the within cluster sum of squares as a measure for the overall similarity within clusters. Visual inspection of the decrease of within cluster sum of squares may or may not provide a useful stopping criterion. In this specific context, however, we suggest an alternative criterion based on the following rationale: From our perspective, it is reasonable to relate the estimated genetic trend in a trait under selection to the trend in inbreeding coefficients. Differences in inbreeding trends between A and G then potentially contribute to a biased trend estimate from Single‐Step. Although in Single‐Step, due to the conditioning of predictions of ungenotyped animals on genotyped animals, part of this bias may be alleviated (Vitezica et al. 2011), adjustment of both inbreeding trends seems to be advisable. The adjustment of structural differences in inbreeding between A and G is one of the fundamental motivations for the modelling of metafounders (Legarra et al. 2015). Therefore, we consider the alignment of inbreeding trends, i.e., of the rate of inbreeding (∆F) in $A_{22}^{\Gamma }$ and G, to be a logical criterion for determining the optimum nMF in the model. However, since the amount of available information may be limited, errors in estimated Γ may accumulate (Legarra et al. 2024a) before a full alignment of inbreeding trends can be achieved. As a supporting criterion, we therefore propose to simultaneously monitor the estimates of the elements of Γ. Diagonals of Γ are self‐relationships of metafounders equaling to two times the inbreeding coefficient and the off‐diagonals of Γ represent relationships between metafounders (Christensen 2012). For both types of elements, valid estimates lie in the range from 0 to 2. Elements outside the parameter space are strong indicators for an accumulation of estimation errors (Legarra et al. 2024a).

Since G is constructed assuming a base allele frequency of 0.5, the average level of inbreeding in G inevitably differs from the unadjusted matrix A. Using a single metafounder (nMF = 1), the mean difference between both matrices is adjusted (Christensen 2012; Vitezica et al. 2011). However, this adjustment does not affect the rate of inbreeding (∆F) in either of these matrices. Recent publications do however indicate that there can be remarkable differences in ∆F depending on whether it is calculated from matrix A **or** G, especially in dairy populations underlying strong genomic selection (Lozada‐Soto et al. 2022). These authors observed higher inbreeding rates from G than from from A. We argue that this observation is at least partially explainable to be a consequence of (structural) information about the pedigree base (contained in G) that is missing when using matrix A based on known pedigrees. By transferring the information on structure among the PF via Γ from genotyped animals to A, a harmonisation of both inbreeding trends can be expected. Therefore, the value of nMF where G and $A^{\Gamma }$ show the most similar rate of inbreeding (∆F) should be optimal. Only genotyped animals (following the study design) were considered for the evaluation of ∆F in both matrices. In either case ∆F was calculated as the slope of ln($1-\bar{F}$) on the birth years of animals (Pérez‐Enciso 1995; Teegen et al. 2009). We chose birth years from 2015 to 2024 to achieve a reasonable group size for each birth year.

### 2.9 Verification of Results

For the verification of the results, we unmasked the GT of all PF (not considered so far in the investigation) and calculated a ‘true’ Γ ($\Gamma_{\text{true}})$ based on the cluster assignments we already had obtained during the optimisation process using imputed gene‐contents. This way, we were able to evaluate the error of estimation in Γ caused by the unavailability of GT of PF and their immediate progeny in real life situations. In a next step, we used $\Gamma_{\text{true}}$ in the estimation of Single‐Step EBV by establishing $H^{\Gamma_{\text{true}}}$ to assess the effect of the inaccuracy in the estimation of Γ on the prediction quality.

## 3 Results

### 3.1 Description of the Population Structure Based on Pedigree Data

**FIGURE 3:**
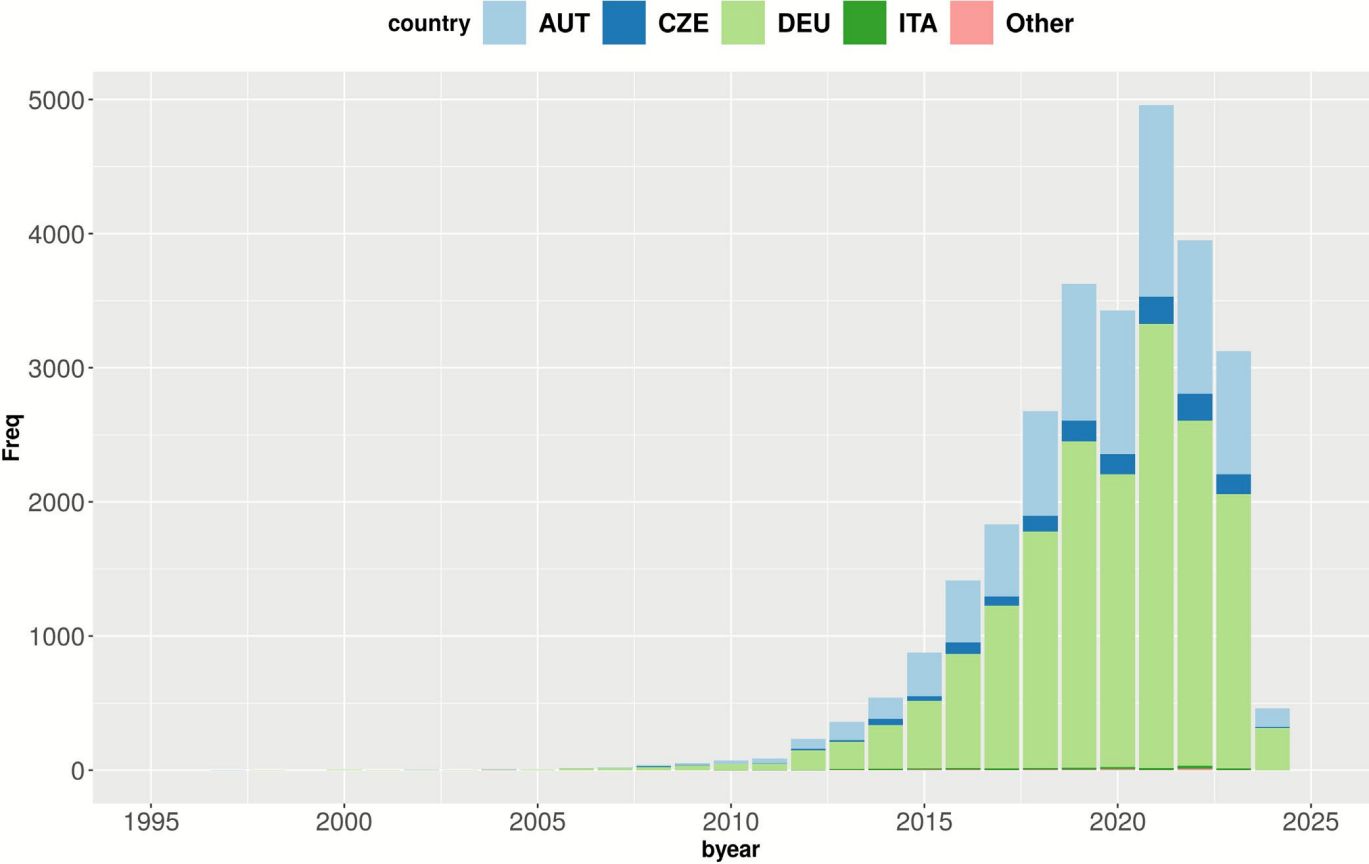
Distribution of all animals in the study population across birth years and countries of origin. [Colour figure can be viewed at wileyonlinelibrary.com]

**FIGURE 4:**
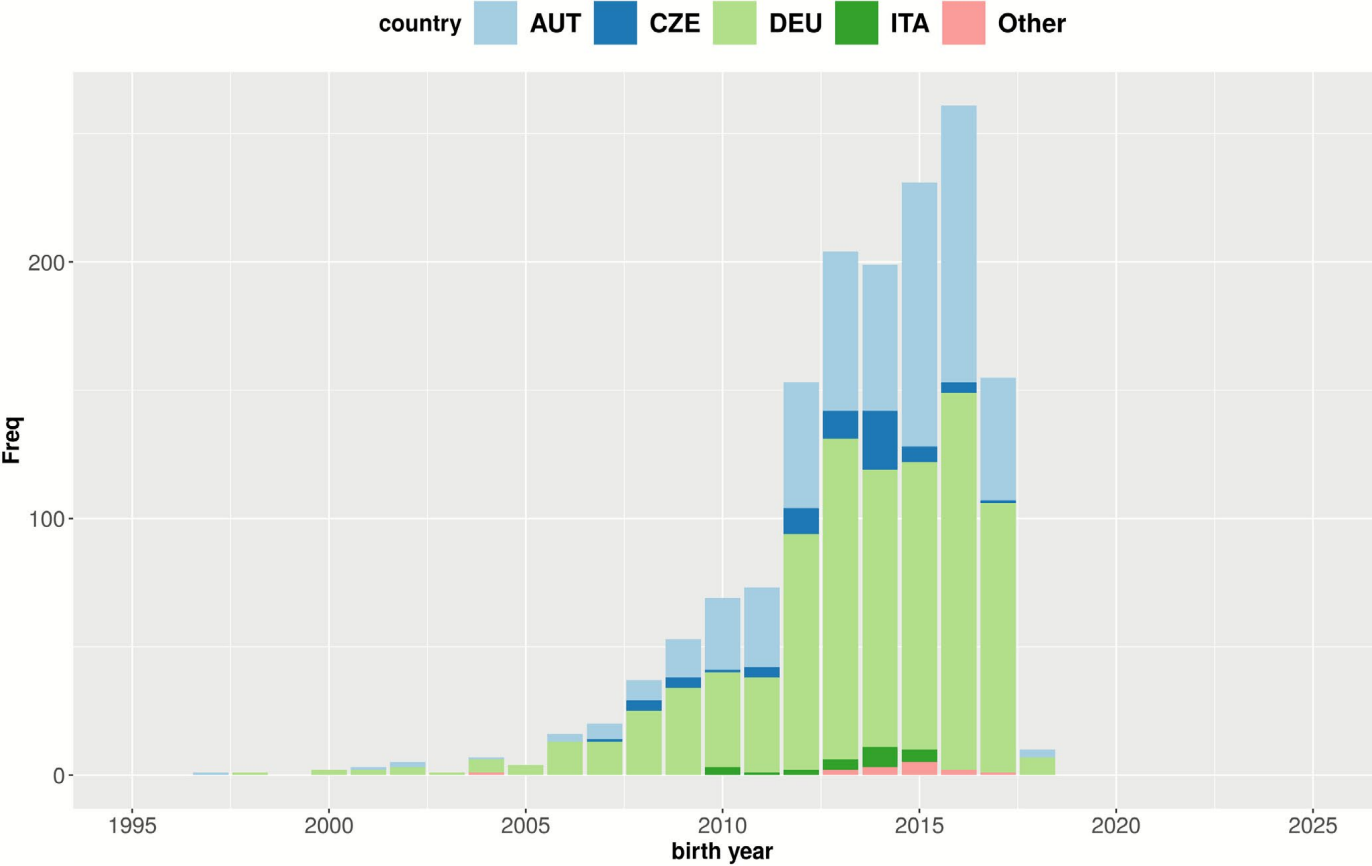
Distribution of pedigree founders in the study population across birth years and countries of origin. [Colour figure can be viewed at wileyonlinelibrary.com]

Statistics on the structure of the study population are given in Tables 2, 3, Figures 3 and 4.

**TABLE 2:** Country of origin of animals in the data set.

Country	All animals	PF
AUT	8297	526
CZE	1258	69
DEU	18,058	873
ITA	139	23
Other	54	14

**TABLE 3:** Pedigree depth study population.

Dataset	Generations traceable								
	0	1	2	3	4	5	6	7	8
PTnoGT	1505	1724	1315	595	255	78	18	0	0
GTnoPT	0	1376	1793	2138	2160	1746	1154	446	13
PTandGT	0	552	1446	2446	2743	2290	1464	542	7

As described in the Methods section, a fully genotyped pedigree of 27,806 related animals was selected for this study. According to ISO‐country code, these animals originated from nine different countries. Germany (DEU) and Austria (AUT) were the largest contributors. Czechia (CZE) was a third important country of origin, followed by Italy (ITA). DEU and AUT can be considered as a single population for the last 20 years. Other countries included France, Hungary, Croatia, Finland and Slovenia (‘Other’ in Table 2). For PF, the pattern was similar, with most animals from DEU and AUT, followed by CZE, ITA and Other.

The animals in the study covered birth years from 1997 to 2024 (Figure 3). Most of the animals were born after 2019 (*n* = 15,922). Birth years of PF spanned 1997 to 2018, with most of them born after 2013 (*n* = 856) (Figure 4). In this study population, a vertical stratification (due to different birth years in the PF) predominated, and only a marginal horizontal stratification was observed (due to different countries of origin) because 1399 of 1505 PF were registered as DEU/AUT.

For 20 animals, pedigrees could be traced for eight generations; the majority of animals could trace their pedigree for three generations (Table 3). Within the study population, one of 16,652 unique sire/dam pairings produced 17 offspring, with the majority (*n* = 13,081) providing only one offspring per pairing. Of 1106 unique sires, one produced 549 offspring, while of 7125 unique dams, one produced 61 offspring.

### 3.2 Derivation of a Stopping Criterion

#### 3.2.1 Kmeans‐Based Statistics

**FIGURE 5:**
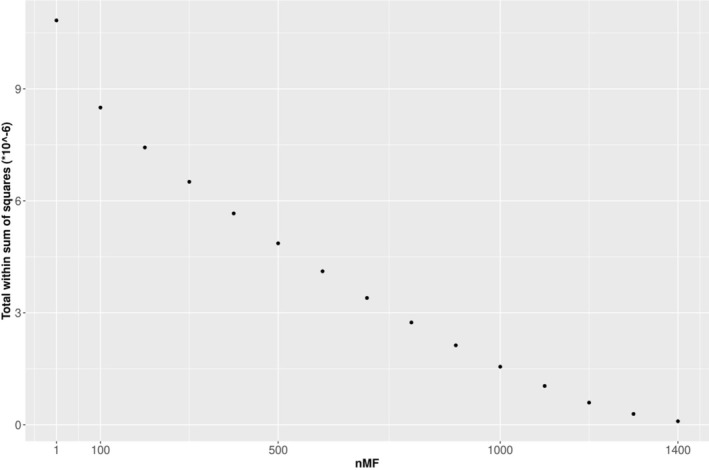
Total within sum of squares for clusters obtained with kmeans.

Total within sum of squares for each nMF cluster are shown in Figure 5. The total within sum of squares continuously decreased with increasing nMF. This kind of analysis did not point to a conclusive optimum nMF.

#### 3.2.2 Based on the Rate in Inbreeding (∆F)

**FIGURE 6:**
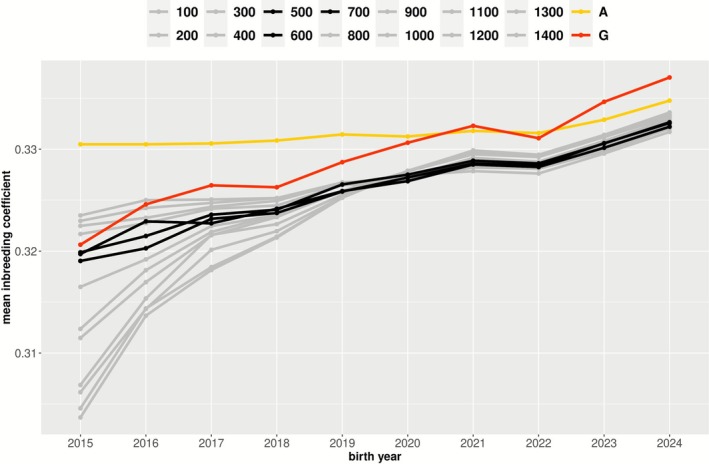
Trend in $\bar{F}$ for birth years 2015 to 2024 for $A^{\Gamma }$ and G. For ease of visualisation, inbreeding coefficients from A were rescaled to the base of G. Trend of F is indifferent to rescaling. [Colour figure can be viewed at wileyonlinelibrary.com]

The trend in mean inbreeding coefficients is shown in Figure 6. The introduction of metafounders affected inbreeding coefficients (F) for all animals. Initially, the mean inbreeding coefficient from $A_{22}$ was 0.002, and for the corresponding G, the mean inbreeding coefficient was 0.331. After rescaling $A_{22}$ using nMF = 1, the (mean) level of inbreeding in $A_{22}^{\Gamma }$ (nMF = 1) was 0.332. Note, that the trend in F was not affected by this rescaling and was considerably different between both matrices (Figure 6). Initial ∆F for G and $A_{22}^{\Gamma }$ (nMF = 1) was 0.00227 and 0.00039, respectively. With the introduction of further structural information (nMF > 1), ∆F for the respective $A_{22}^{\Gamma }$ became more and more similar to ∆F from G. Estimates of ∆F were very close in the range of nMF between 500 and 700, closest values were observed for nMF = 688 (∆F = 0.00224), confirmed by visual inspection of the $\bar{F}$ of birth years 2015 to 2024. For nMF ≥ 800 ∆F was overestimated in the respective $A_{22}^{\Gamma }$ (∆F ≥ 0.00247) (Figure 6).

#### 3.2.3 Based on Properties of Γ

Summary statistics of diagonal and off‐diagonal values of Γ are given in Table 4. The maximum value for diagonals of Γ increased with increasing nMF, as did the mean and standard deviation. The minimum value showed a slight decrease from nMF = 1 to nMF = 100 with continuous increase for nMF > 200. Starting with nMF = 700, the first diagonal element of Γ outside the parameter space occurred (Table 4). For nMF ≥ 700, we observed an increasing range of diagonal elements, and more and more estimated diagonals were outside of the parameter space. Analysing off‐diagonal values showed a similar behaviour, with an increase in maximum value and range. For nMF ≥ 1000, off‐diagonals below 0 and for nMF ≥ 1300, maximum values larger than 2 were observed (Table 4).

**TABLE 4:** Analysis of diagonal elements of Γ. This table gives a summary statistic of diagonal elements of Γ, with the number of elements out of parameter space (> 2, N out).

nMF	Diagonal					Off‐diagonal			
	Min	Mean	Max	SD	N out	Min	Mean	Max	N out
1	0.650	0.650	0.650	NA		NA	NA	NA	
100	0.643	0.959	1.463	0.133		0.560	0.647	0.819	
200	0.643	1.106	1.627	0.201		0.560	0.647	0.884	
300	0.646	1.201	1.646	0.217		0.548	0.648	0.912	
400	0.649	1.295	1.923	0.232		0.511	0.648	0.930	
500	0.650	1.352	1.922	0.225		0.236	0.649	0.971	
600	0.650	1.405	1.980	0.220		0.475	0.648	1.047	
700	0.652	1.458	2.079	0.223	1	0.421	0.648	1.021	
800	0.652	1.508	2.335	0.227	10	0.422	0.647	1.047	
900	0.655	1.564	2.396	0.239	37	0.008	0.647	1.051	
1000	0.658	1.625	2.872	0.269	93	−0.204	0.647	1.252	2
1100	0.661	1.694	2.873	0.310	180	−0.204	0.647	1.253	3
1200	0.666	1.781	3.625	0.378	292	−0.521	0.646	1.253	7
1300	0.689	1.942	6.565	0.644	414	−2.621	0.646	2.225	25
1400	0.844	2.221	9.933	1.148	527	−3.657	0.645	2.964	57

Other evaluation criteria (i.e., difference in total mean and mean diagonal of G and $A^{\Gamma }$) showed a continuous improvement up to the maximum nMF modelled (results not shown) and gave no conclusive optimum.

### 3.3 Effect of Metafounders on EBV

**FIGURE 7:**
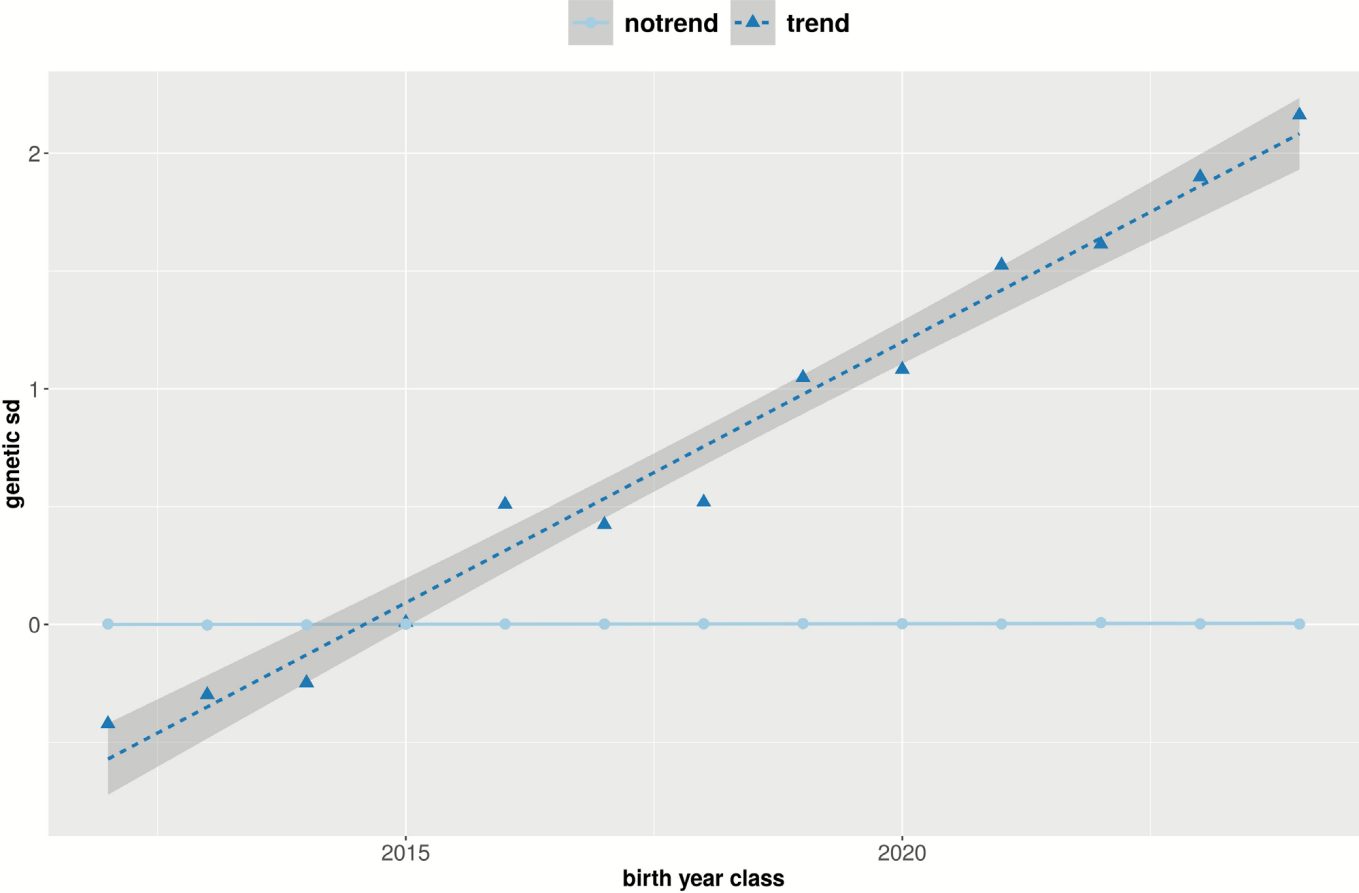
Trend in mean TBV in a trait with trend (dark blue dotted line and triangles) or without trend (light blue line and dots). Shaded area represents the 95% CI. For each animal, mean TBV was calculated as the mean of 500 repeats, triangles and dots represent mean of each birth year class of animal specific mean TBV. [Colour figure can be viewed at wileyonlinelibrary.com]

The trend in TBV is shown in Figure 7 for scenario ‘trend’ and ‘notrend’. Model parameters are given in Table 5 for both scenarios and results of the optimisation for both scenarios are given in Table 6. Results are means of 500 repetitions of sampling gene effects.

**TABLE 5:** Displayed are the coefficient matrix (coef) used in the model, nMF, τ, $\sigma_{a_{\text{resc}}}^{2}$, $h_{\text{resc}}^{2}$, for a case without genomic information (coef = **A**) and the case with genomic information and modelling of metafounders (coef = **H** ^**Γ**^) for both scenarios trend and notrend. The model parameters, $\sigma_{a_{\text{resc}}}^{2}$, $h_{\text{resc}}^{2}$ are the same for all groups and only differ between scenarios and nMF modelled.

Coef	nMF	Trend			Notrend		
		τ	$\sigma_{a_{\text{resc}}}^{2}$	$h_{\text{resc}}^{2}$	τ	$\sigma_{a_{\text{resc}}}^{2}$	$h_{\text{resc}}^{2}$
**A**	0	0.000	1.000	0.250	0.000	1.000	0.250
**H** ^Γ^	1	0.650	1.481	0.330	0.651	1.483	0.331
	100	0.341	1.206	0.287	0.339	1.204	0.286
	200	0.194	1.107	0.270	0.207	1.115	0.271
	400	0.005	1.003	0.250	0.019	1.010	0.252
	600	−0.106	0.950	0.240	−0.098	0.953	0.241
	800	−0.211	0.905	0.232	−0.207	0.906	0.232
	1000	−0.330	0.858	0.222	−0.325	0.860	0.223
	1200	−0.486	0.805	0.211	−0.486	0.805	0.212
	1400	−0.928	0.683	0.186	−0.926	0.684	0.186

**TABLE 6:** Displayed are the coefficient matrix (coef) used in the model, nMF, Intercept (a), regression coefficient (b) and fit (*R* ^2^) of the regression of TBV on EBV and the bias (TBV‐EBV) for a case without genomic information (coef = **A**) and the case with genomic information and modelling of metafounders (coef = **H** ^**Γ**^) for all groups of animals for specific nMF for both scenarios trend and notrend. Bias and a are displayed in an additive genetic standard deviation (set for PF). Results presented here are means of 500 repetitions of sampling of gene effects, i.e., each animal obtained 500 TBV, 500 phenotypes and 500 GEBVs. Symbols in superscript relate to the standard deviation (see footnote).

Dataset	Coef	nMF	Trend				Notrend			
			*a* ^b^	*b* ^a^	*R* ^2,^ ^a^	Bias^a^	*a* ^b^	*b* ^b^	*R* ^2,^ ^b^	Bias^a^
All	**A**	0	0.060	0.955	0.683	−0.011	0.001	1.000	0.400	−0.001
	**H** ^**Γ**^	1	0.104	0.913	0.763	−0.007	0.001	0.991	0.559	−0.001
		100	0.068	0.945	0.776	−0.006	0.001	1.046	0.558	−0.001
		200	0.054	0.957	0.780	−0.007	0.001	1.066	0.557	−0.001
		400	0.040	0.970	0.784	−0.007	0.001	1.092	0.554	−0.001
		600	0.031	0.978	0.785	−0.007	0.001	1.107	0.552	−0.001
		800	0.024	0.984	0.786	−0.007	0.001	1.119	0.550	−0.001
		1000	0.017	0.991	0.787	−0.007	0.001	1.131	0.546	−0.001
		1200	0.010	0.998	0.786	−0.007	0.001	1.144	0.540	−0.001
		1400	−0.009	1.016	0.784	−0.009	0.001	1.174	0.525	−0.001
GTnoPT	**A**	0	0.043	0.986	0.674	−0.026	0.001	0.997	0.316	−0.001
	**H** ^**Γ**^	1	0.132	0.903	0.757	−0.018	0.001	0.981	0.533	−0.001
		100	0.088	0.939	0.767	−0.016	0.001	1.035	0.532	−0.001
		200	0.073	0.951	0.771	−0.016	0.001	1.055	0.532	−0.001
		400	0.058	0.965	0.774	−0.017	0.001	1.081	0.530	−0.001
		600	0.048	0.974	0.776	−0.017	0.001	1.097	0.529	−0.001
		800	0.038	0.982	0.778	−0.017	0.001	1.112	0.527	−0.001
		1000	0.029	0.990	0.779	−0.017	0.001	1.127	0.525	−0.001
		1200	0.016	1.001	0.780	−0.018	0.001	1.148	0.522	−0.001
		1400	−0.013	1.029	0.782	−0.021	0.001	1.200	0.513	−0.001
PTandGT	**A**	0	0.153	0.902	0.666	−0.024	0.001	1.002	0.451	−0.001
	**H** ^**Γ**^	1	0.167	0.885	0.754	−0.015	0.001	0.994	0.600	−0.001
		100	0.122	0.920	0.765	−0.016	0.001	1.048	0.599	−0.001
		200	0.106	0.933	0.769	−0.017	0.001	1.068	0.597	−0.001
		400	0.087	0.948	0.774	−0.018	0.001	1.095	0.595	−0.001
		600	0.076	0.956	0.776	−0.019	0.001	1.111	0.594	−0.001
		800	0.066	0.964	0.777	−0.019	0.000	1.127	0.592	−0.001
		1000	0.056	0.973	0.779	−0.020	0.000	1.143	0.590	−0.001
		1200	0.042	0.985	0.780	−0.022	0.000	1.165	0.586	−0.001
		1400	0.009	1.013	0.783	−0.026	0.000	1.218	0.576	−0.001
PTnoGT	**A**	0	−0.016	0.934	0.595	0.048	0.001	1.000	0.458	−0.001
	**H** ^**Γ**^	1	0.007	0.925	0.683	0.029	0.001	1.004	0.523	−0.001
		100	−0.007	0.951	0.710	0.030	0.001	1.062	0.522	−0.001
		200	−0.016	0.966	0.714	0.032	0.001	1.082	0.520	−0.001
		400	−0.026	0.980	0.719	0.035	0.001	1.107	0.514	−0.001
		600	−0.030	0.984	0.720	0.037	0.001	1.115	0.509	−0.001
		800	−0.031	0.986	0.720	0.038	0.001	1.117	0.503	−0.001
		1000	−0.032	0.984	0.716	0.040	0.001	1.111	0.495	−0.001
		1200	−0.030	0.974	0.708	0.043	0.001	1.089	0.481	−0.001
		1400	−0.028	0.952	0.687	0.052	0.001	1.044	0.447	−0.001

^a^ SE ≤ 0.001.

^b^ SE ≤ 0.0015.

#### 3.3.1 Scenario ‘trend’

A strong genetic trend was generated, corresponding to an increase of about 1 genetic standard deviation in 5 years (Figure 7). The mean TBV was negative before birth year 2015, which roughly corresponds to the average birth year of the PF (Figures 3 and 4).

**FIGURE 8:**
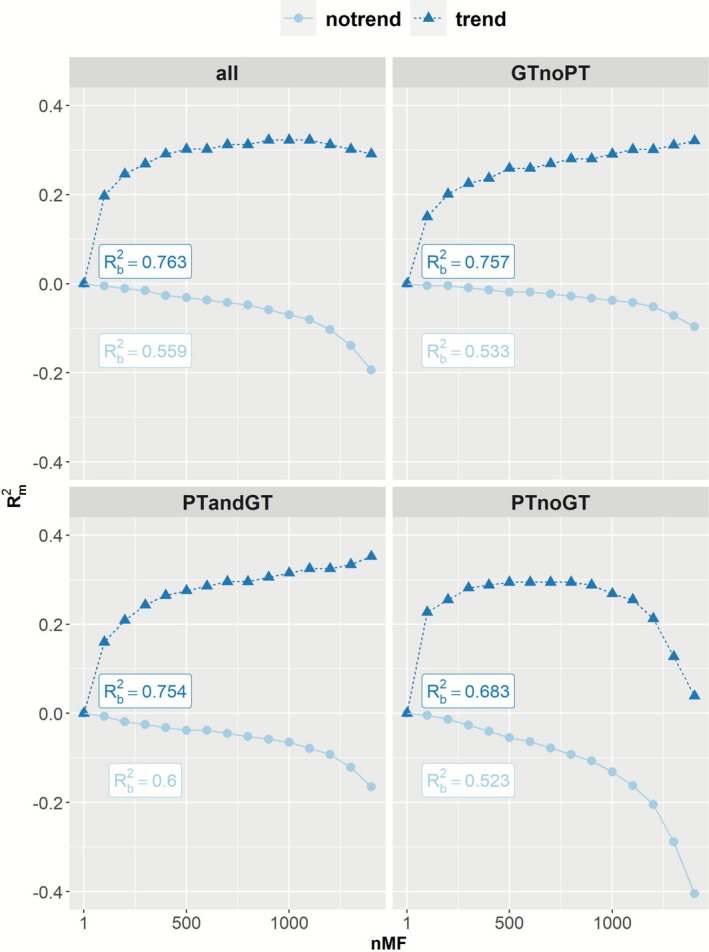
Results ($R_{m}^{2}$) of the optimisation process for different groups of animals for all nMF considered. *R* ^2^ for nMF = 1 (*R*_b_^2^) for the respective groups are: 0.763, 0.757, 0.754 and 0.683 for all, GTnoPT, PTandGT and PTnoGT for scenario trend. For scenario without trend the respective *R*_b_^2^ are: 0.559, 0.533, 0.600, and 0.523 for all, GTnoPT, PTandGT and PTnoGT. [Colour figure can be viewed at wileyonlinelibrary.com]

**FIGURE 9:**
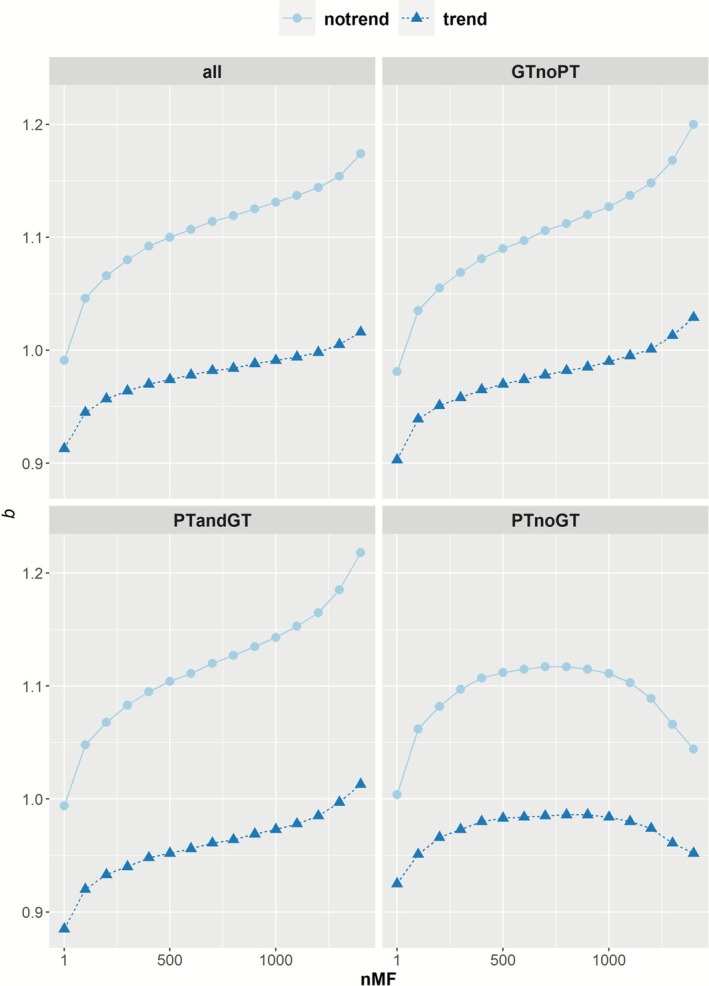
Results (b) of the optimisation process for different groups of animals for all nMF considered for scenario with trend (dark blue dotted line and triangles) and without trend (light blue solid line and dots). [Colour figure can be viewed at wileyonlinelibrary.com]

The results of the optimisation process for this scenario are given in Table 6, Figures 8 and 9. In general, an increase in the number of metafounders improved *R* ^2^ (Figure 8, Table 6). We investigated smaller numbers of metafounders (nMF < 100) but found little improvement in prediction quality gained by small steps of increasing nMF (results not shown). Increase in *R* ^2^ for all animals reached a plateau for values of nMF between 600 and 1000. Behaviour for *R* ^2^ was similar for genotyped animals (GTnoPT and PTandGT). For non‐genotyped animals (PTnoGT), a clear plateau was observed for values of nMF between 400 and 800 (Figure 8) with a strong decrease afterwards. For groups GTnoPT and PTandGT, a slight increase in *R* ^2^ was observable even for nMF ≥ 1000. The largest single increase in *R* ^2^ was observed from nMF = 1 to 100 with diminishing marginal increases for nMF > 100 (Figure 8). Standard deviations for *R* ^2^ were in the range of 0.0003–0.0005 (Table 6).

Slope of regression for standard Single‐Step (nMF = 1) was smaller than for a model based only on A (b = 0.913 vs. b = 0.955). With increasing values of nMF the b's approached 1 in a continuous manner. Behaviour was similar for all genotyped groups (GTnoPT and PTandGT). Again, group PTnoGT showed a more definite behaviour by approaching a plateau for nMF = 400 to 1000, with steady decrease afterwards. Calculated across all groups, the estimated slopes were between 0.97 and 1.00 in the range of nMF = 400 to 1200 (Figure 9). Standard deviations for b were in the range of 0.0005 to 0.0007 (Table 6).

In all analysed groups, the actual bias calculated as the average of EBV minus TBV was negligible, never exceeding 0.05 of an additive genetic standard deviation throughout the optimisation process. Standard deviations for bias were in the range of 0.0007–0.0008 (Table 6). Estimated intercepts (*a*) were highest for nMF = 1 in groups assigned to be genotyped with values in the range of 0.1 of an additive genetic standard deviation and decreased continuously for increasing nMF except for group PTnoGT where the intercept was approximately 0 for all values of nMF (Table 6). Standard deviations for a were in the range of 0.0008 to 0.0011 (Table 6). Concerning the reranking of animals for different nMF, we calculated the Pearson correlation coefficient among EBV between nMF = 1 and all other nMF. The correlation among EBV decreased linearly with increasing nMF, starting at 0.998 between nMF = 1 and nMF = 100 and reaching 0.982 between nMF = 1 and nMF = 1400.

#### 3.3.2 Scenario ‘Notrend’

The introduction of genomic information by using a standard Single‐Step model (nMF = 1) led to an increase in *R* ^2^ for all groups (Table 6). Modelling more metafounders did not improve *R* ^2^ any further. In absolute values, we observed a slight decrease from nMF = 1 to 1400 in groups all, GTnoPT and PTandGT (Table 6). The group GTnoPT was least affected. Results for group PTnoGT were affected the most and a decrease in *R* ^2^ was especially pronounced for nMF > 1000. Standard deviations for *R* ^2^ were in the range of 0.0007 to 0.0011 (Table 6).

For all groups of animals considered, EBVs and standard Single‐Step EBVs (nMF = 1) were unbiased (*b* ≈ 1) (Table 6). With increasing values of nMF, b's increased for all groups indicating a growing deflation of EBV. Similar to the behaviour of PTnoGT in the scenario ‘trend’, we observed an approach to a plateau (nMF = 400 to 1000) and a decreasing deflation of EBV afterwards (Figure 9). All groups showed a similar shape of the trends in b's to the one observed from scenario with trend. Standard deviations for b were in the range of 0.0007 to 0.0014 (Table 6).

Bias and a were not affected by the modelling of metafounders in the ‘notrend’ scenario and remained stable at very low levels for all nMF and groups (Table 6). Standard deviations for a were in the range of 0.0006 to 0.0012 and in the range of 0.0006 to 0.0008 for bias (Table 6).

### 3.4 Aspects of Rescaling the Additive‐Genetic Variance

In our study design, the variance of the generated TBV of PF was rescaled to give $\sigma_{a}^{2}$ = 1 and an appropriate residual variance was chosen to achieve an *h* ^2^ of 0.25 in the pedigree base. These were the parameters applied in the conventional animal model used as a base‐line scenario (covariance structure was A, as depicted in Table 5). Introducing nMF = 1 and establishing the Single‐Step model, matrix A and the additive‐genetic variance had to be rescaled to match the genomic relationship matrix G built on the assumption of a uniform base allele frequency of 0.5. This fundamental rescaling led to estimates of $h_{\text{resc}}^{2}$ = 0.330 and $\sigma_{a_{\text{resc}}}^{2}$ = 1.481 for scenario ‘trend’ (Table 5). Introducing structural information by further increasing nMF resulted in continuously decreasing values for adjusted $\sigma_{a_{\text{resc}}}^{2}$ and $h_{\text{resc}}^{2}$. For nMF ≥ 600, we observed negative values for τ. All these observations apply equally in scenarios with or without genetic trend, since these adjustments rely on properties of Γ as estimated from genotyped animals only. There was, however, a small difference in Γ estimates for the two scenarios, which can be explained by the different SNPs chosen to be QTL that were masked after the generation of TBV.

### 3.5 Verification of Results (Using True Genotypes of Founders)

#### 3.5.1 Comparing the Elements of Γ

Results of the comparison between Γ and $\Gamma_{\text{true}}$ are available from Table 7. For $\Gamma_{\text{true}}$, no values outside the parameter space were observed. For diagonal elements we observed an increasing difference in mean value and variance between the two matrices (Table 7), indicating an overestimation in Γ. For nMF ≥ 600, this overestimation started to become more severe, with the variance observed in diagonal elements of Γ twice as high as observed in $\Gamma_{\text{true}}$. In analyses of off‐diagonal values, only minor differences in Γ and $\Gamma_{\text{true}}$ were found (results not shown).

**TABLE 7:** Deviation of diagonal elements of Γ from diagonal elements of $\Gamma_{\text{true}}$ in mean (absolute difference) and variance ($\mathrm{Var}\left(\Gamma \right)/\mathrm{Var}\left(\Gamma_{\text{true}}\right)$). For nMF = 1 Γ is a scalar.

nMF	Absolute difference in mean	$\mathrm{Var}\left(\Gamma \right)/\mathrm{Var}\left(\Gamma_{\text{true}}\right)$
1	0.005	NA
100	0.041	1.278
200	0.076	1.475
300	0.099	1.453
400	0.132	1.703
500	0.157	1.844
600	0.185	2.006
700	0.219	2.428
800	0.253	3.050
900	0.292	4.112
1000	0.342	6.402
1100	0.400	11.078
1200	0.479	23.403
1300	0.636	84.725
1400	0.911	392.035

#### 3.5.2 Effects on EBV When Using $\Gamma_{\text{true}}$

**FIGURE 10:**
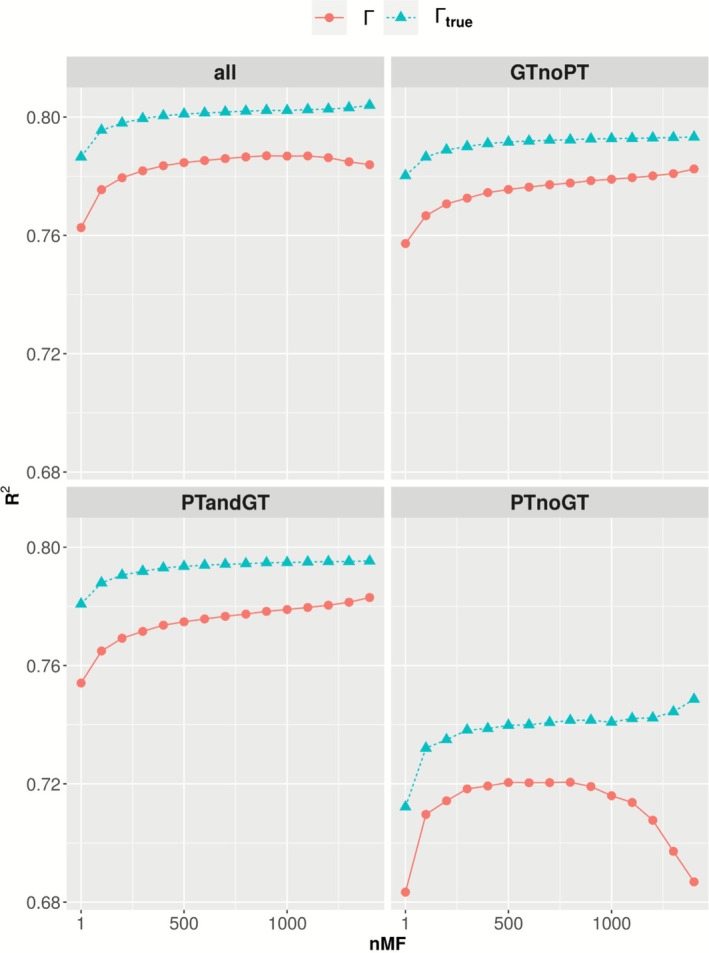
Results (*R* ^2^) of the optimisation process for different groups of animals for all nMF considered in the scenario with trend for Γ from GT observed of PF (blue dashed line with triangles) and Γ from estimated metafounder allele frequencies (red solid line with dots). [Colour figure can be viewed at wileyonlinelibrary.com]

**FIGURE 11:**
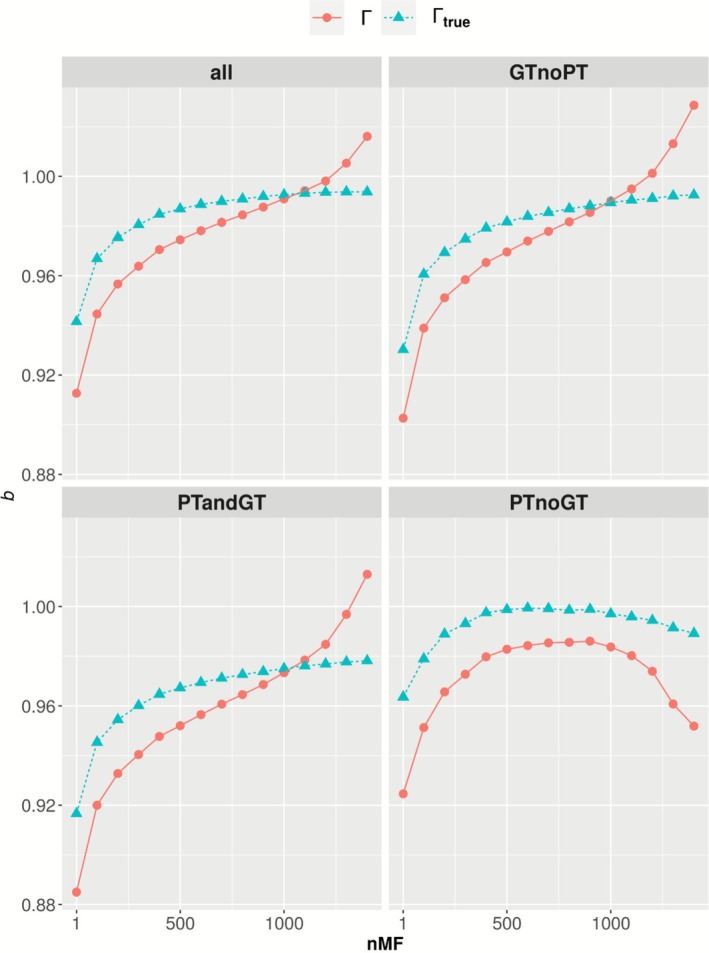
Results (b) of the optimisation process for different groups of animals for all nMF considered in the scenario with trend for Γ from GT observed of PF (blue dashed line with triangles) and Γ from estimated metafounder allele frequencies (red solid line with dots). [Colour figure can be viewed at wileyonlinelibrary.com]

For the sake of brevity, we only show results for the scenario with a strong genetic trend for this comparison in Figures 10, 11 and Table 8. For all groups, we obtained slightly higher *R* ^2^ using $\Gamma_{\text{true}}$, compared to values observed using Γ (Figure 10, Table 8). The curves for groups GTnoPT and PTandGT were very similar to those obtained with Γ. However, for group PTnoGT no plateau for *R* ^2^ was observed with $\Gamma_{\text{true}}$.

**TABLE 8:** Results for scenario with trend and $\Gamma_{\text{true}}$. Displayed are the coefficient matrix (coef) used in the model, nMF, $\sigma_{a_{\text{resc}}}^{2}$, $h_{\text{resc}}^{2}$, intercept (a), regression coefficient (b) and fit (*R* ^2^) of the regression of TBV on EBV and the bias (TBV‐EBV) for a case without genomic information (coef = **A**) and the case with genomic information and modelling of metafounders (coef = **H** ^**Γ**^) for all groups of animals for specific nMF. Bias and a are displayed in an additive genetic standard deviation (set for PF).

Dataset	Coef	nMF	τ	$\sigma_{a_{\text{resc}}}^{2}$	$h_{\text{resc}}^{2}$	*a*	*b*	*R* ^2^	Bias
All	**H** ^**Γ**^	1	0.645	1.476	0.330	0.072	0.942	0.787	−0.007
		200	0.267	1.154	0.278	0.034	0.975	0.798	−0.007
		400	0.135	1.072	0.263	0.024	0.985	0.800	−0.007
		600	0.077	1.040	0.257	0.019	0.989	0.801	−0.007
		800	0.040	1.021	0.254	0.017	0.991	0.802	−0.007
		1000	0.012	1.006	0.251	0.015	0.993	0.802	−0.007
		1200	−0.009	0.996	0.249	0.014	0.994	0.803	−0.007
		1400	−0.018	0.991	0.248	0.014	0.994	0.804	−0.007
GTnoPT	**H** ^**Γ**^	1	0.645	1.476	0.330	0.100	0.930	0.780	−0.018
		200	0.267	1.154	0.278	0.052	0.969	0.789	−0.016
		400	0.135	1.072	0.263	0.041	0.979	0.791	−0.017
		600	0.077	1.040	0.257	0.035	0.984	0.792	−0.017
		800	0.040	1.021	0.254	0.032	0.987	0.792	−0.016
		1000	0.012	1.006	0.251	0.029	0.989	0.793	−0.016
		1200	−0.009	0.996	0.249	0.027	0.991	0.793	−0.017
		1400	−0.018	0.991	0.248	0.025	0.993	0.793	−0.017
PTandGT	**H** ^**Γ**^	1	0.645	1.476	0.330	0.125	0.917	0.781	−0.015
		200	0.267	1.154	0.278	0.077	0.954	0.791	−0.016
		400	0.135	1.072	0.263	0.064	0.965	0.793	−0.017
		600	0.077	1.040	0.257	0.058	0.969	0.794	−0.018
		800	0.040	1.021	0.254	0.054	0.973	0.794	−0.018
		1000	0.012	1.006	0.251	0.051	0.975	0.795	−0.018
		1200	−0.009	0.996	0.249	0.049	0.977	0.795	−0.018
		1400	−0.018	0.991	0.248	0.047	0.978	0.795	−0.018
PTnoGT	**H** ^**Γ**^	1	0.645	1.476	0.330	−0.012	0.964	0.712	0.029
		200	0.267	1.154	0.278	−0.027	0.989	0.735	0.032
		400	0.135	1.072	0.263	−0.033	0.998	0.739	0.034
		600	0.077	1.040	0.257	−0.034	0.999	0.740	0.035
		800	0.040	1.021	0.254	−0.034	0.999	0.741	0.035
		1000	0.012	1.006	0.251	−0.034	0.997	0.741	0.035
		1200	−0.009	0.996	0.249	−0.033	0.994	0.742	0.036
		1400	−0.018	0.991	0.248	−0.030	0.989	0.749	0.035

With $\Gamma_{\text{true}}$, EBVs were slightly less inflated for nMF ≤ 1000(Figure 11, Table 8) and showed an asymptotic trend towards 1 for nMF ≥ 600.

Intercept and bias were small and never exceeded 0.09 and 0.04 of an additive genetic standard deviation, respectively (Table 8).

For the rescaling of the additive‐genetic variance, we observed a similar behaviour as in the optimisation process, with an initial increase for nMF = 1 ($\sigma_{a_{\text{resc}}}^{2}$ = 1.476 and $h_{\text{resc}}^{2}$ = 0.330) followed by a continuous decrease. Notably even with $\Gamma_{\text{true}}$, τ was negative for nMF ≥ 1200 (Table 8).

## 4 Discussion

In this study, we used a semi‐stochastic simulation based on real Fleckvieh genotypes to test an approach of identifying metafounders by a standard clustering algorithm based on imputed GT of PF. For the population we investigated, identification of metafounders based on this approach was possible and improved prediction quality for a trait with a strong genetic trend. The proposed approach provides a way to reconstruct the true relationship among pedigree founders from ‘imputed’ genotypes. We additionally propose a combination of monitoring of ∆F estimates and of elements of Γ to identify an optimum number of metafounders in practical applications.

### 4.1 Study Population

Because of the strategies involved in selecting the animals of the study population and our approach to assign them to be either ‘genotyped’, ‘phenotyped’ or both, the information content in the study population was rather high and with respect to several aspects idealistic. For example, although not providing GT themselves, all PF were connected via pedigree to the genotyped population and were only a distance of two generations away from genotyped animals. This short distance likely facilitated the estimation of informative gene‐contents for the clustering algorithm as well as the estimation of Γ, the self‐relationship of metafounders. In this study population stratification occurred predominantly in a vertical manner because of missing pedigree information. As a result, the pedigree base spanned over several generations and it was in this respect, that the pedigree base was not homogeneous. Investigation of country code of selected animals suggested that stratification due to different country origins was of secondary importance for the population investigated. This is quite a typical situation for dairy cattle, where a population is frequently characterised by a main population and the occasional introgression of other breeds or from animals of the same breed from other breeding‐programs.

### 4.2 Derivation of a Stopping Criterion

Any practical application of our approach requires an objective stopping criterion. Analysing within cluster sums of squares did not lead to conclusive results, as with our data we observed no ‘elbow’ shape, which is often used as an indicator for an optimum number of clusters (e.g., (Himmelbauer et al. 2024)). As a conclusion of our investigation, we suggest a combined strategy that primarily aims at harmonising the rate of inbreeding observed in G and $A_{22}^{\Gamma }$. How successful this harmonisation is, critically depends on the identification of truly relevant metafounders which in turn depends on the global informativity of the system. In other words, the accuracy of the elements of Γ is conditional to the number of genotyped animals in the population and their connectedness to the PF. Consequently, the harmonisation of ∆F might not be fully achievable before errors in the estimation of Γ accumulate. We therefore recommend to additionally monitor the estimated elements of Γ and to stop the optimisation if a considerable (e.g., 20%) number of estimates are found to be out‐of‐parameter‐space (0 to 2 for elements of Γ).

In contrast to assumptions of standard quantitative‐genetic theory, where inbreeding is not intended to describe allele‐frequency changes but an increasing deviation from HW‐proportions at neutral loci, the trend observed in genomic inbreeding coefficients does reflect non‐random changes in allele frequencies. It therefore exhibits the effect of selective processes on allele frequencies and is capable of modeling the corresponding changes in the mean of a trait that responds to selection (Anglhuber et al., in prep; Falconer and Mackay 2009). This observation was also determining the way we simulated the TBV in the scenario with trend.

The resulting accumulation of the global homozygosity at genomic markers is described by the genomic inbreeding coefficients if matrix G is constructed with the assumption of a uniform base‐allele frequency of 0.5 across all markers (Anglhuber et al. 2024). It has been frequently reported that inbreeding coefficients derived from matrix A not only show a relatively weak correlation to this genomic inbreeding but also that the rate of inbreeding observed from both matrices showed considerable differences (Lozada‐Soto et al. 2022). This implies that in the estimation of EBV a conventional and a genomic evaluation model regress the genetic trend observed on different independent variables (the inbreeding coefficients from both systems). When combining both matrices in a single model this difference can contribute to inflated trend estimates. Several approaches were proposed and are used in practice to align the mean inbreeding of G and $A_{22}$ (Christensen 2012; Forni et al. 2011; Vitezica et al. 2011; Meuwissen et al. 2011). This type of rescaling does, however, not affect the difference in ∆F estimates of both matrices. As we have demonstrated in this investigation, it is possible to increase the similarity between the inbreeding trends in both matrices and to simultaneously reduce the inflation of EBV derived from the Single‐Step model by increasing the number of metafounders in the model.

Diagonal elements of Γ are sensitive to errors in the estimation process, since they are quadratic functions of the estimates of metafounder allele frequencies (Legarra et al. 2024a). First diagonal estimates outside the parameter space were observed from nMF = 700 onwards which roughly coincides with the nMF that achieves similar ∆F in G and $A_{22}^{\Gamma }$. Assessing the accuracy of the estimated elements of Γ by counting the number of out‐of‐parameter‐space estimates obviously is not an ideal criterion since global accuracies might deteriorate long before the first invalid estimates appear. On the other hand, single out‐of‐parameter‐space estimates for metafounders with very little information available to estimate their allele frequencies may occur although the majority of relevant metafounders are still estimated with sufficient accuracy. Perhaps a weighted criterion additionally considering the impact of each metafounder on the population (i.e., number of genotyped animals representing a specific metafounder) would contribute to a more concise picture but this was beyond what reasonably could be inferred from the simulated example used here.

**FIGURE 12:**
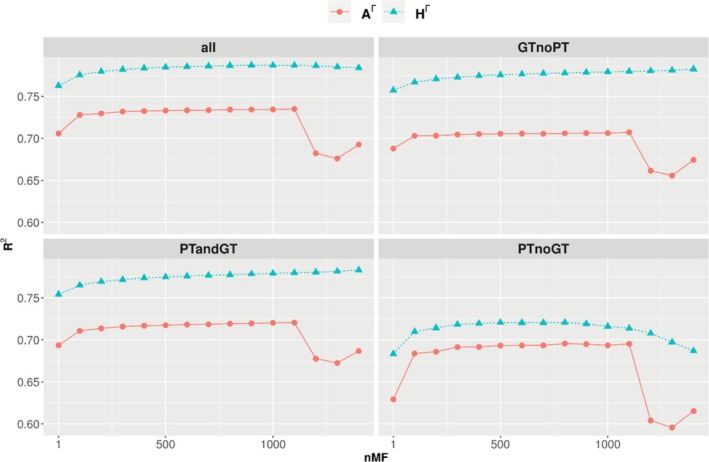
Comparison of *R* ^2^ obtained from a model based only on $A^{\Gamma }$ (red solid line and dots) to *R* ^2^ from the optimisation process (blue dotted line and triangles). [Colour figure can be viewed at wileyonlinelibrary.com]

It is worth to emphasise that although the accuracy of the estimated elements of Γ decreased with nMF ≥ 1000 (Table 4), results from the estimation on Single‐Step EBV were stable for a trait with a strong genetic trend (Figure 8, Table 6). Only group PTnoGT, which included the PF and therefore animals closest to the metafounders, showed a different behaviour. For the youngest animals (GTnoPT), which are of most interest in populations under selection (selection candidates), results were stable even in this range of nMF and beyond. To get a clearer picture we repeated the whole optimisation solely using $A^{\Gamma }$ for the prediction of EBV and steadily increasing nMF. The observed *R* ^2^ showed a similar behaviour as observed with $H^{\Gamma }$ for nMF < 1200. With nMF ≥ 1200 however, *R* ^2^ decreased rapidly in all groups (Figure 12). This suggests that the genomic information and the way it is utilised in setting up $H^{\Gamma }$ balances out errors in Γ to some degree. Nevertheless, we recommend a close monitoring of elements in Γ in addition to the monitoring of ∆F to identify the optimum nMF.

### 4.3 Effect of Metafounders on EBV

For the scenario with a strong genetic trend standard Single‐Step (nMF = 1) was the least reliable model among all Single‐Step models and showed a moderate trend bias (b = 0.913). The introduction of nMF > 1 considerably improved prediction quality. Maximum *R* ^2^ occurred in the range between nMF = 500 and nMF = 1000, where also predictions virtually showed no trend‐bias. The slope of regression was better in this range than for nMF = 1, but it increased even further with increasing nMF. For nMF > 1000 overall prediction quality deteriorated in the group of animals providing phenotypes but no genotypes (PTnoGT). The other groups showed slight increases even for nMF > 1000. The largest improvement in all groups occurred in the step from nMF = 1 to nMF = 100. Since the overall number of PF in the study population was 1505, the optimum for nMF was in a range where the structural decomposition of the pedigree base was already very fine‐grained and the prediction quality for the elements of Γ clearly showed indications for an increasing error rate (nMF = 700 onward). However, no corresponding effect on the quality of the EBV was observable until nMF = 1000. This suggests that EBV predictions are relatively robust against the errors in Γ even with very large numbers of nMF under the conditions observed in the study population. On the other hand, even with nMF = 100 (still a large number considering the size of the pedigree base) a first large improvement can be achieved. Considering the allele‐frequency changes characterising the genomic data used and the method we used to generate the genetic trend we conclude that metafounders in this scenario considerably helped to model the genetic trend by compensating the difference in birth year means and therefore improve prediction quality even if the underlying structure is not fully uncovered.

For the ‘notrend’ scenario, the solutions using standard Single‐Step (nMF = 1) where the most reliable and showed no trend bias (*b* = 0.991). With increasing nMF we observed a slight reduction in *R* ^2^ (Figure 8, Table 6). This slight decrease in prediction quality can be expected, since in the absence of a (strong) genetic trend the model tries to fit differences between metafounder means that do not exist. This adds unnecessary complexity to the model and thus reduces the prediction quality. Similar results were reported by Bradford et al. (2019) in a simulation study with UPG. In our study, reductions in *R* ^2^ remained acceptably low up to nMF ~ 700. We conclude that the consideration whether to model metafounders or not does not necessarily need to be conditional on the presence of a strong genetic trend in a specific trait. However, with no genetic trend, the unnecessary correction of the parameter of the additive‐genetic variance additionally causes a growing deflation of EBV with increasing nMF (Figure 9, Table 6).

The scenario without genetic trend was included to investigate a case where the allele frequency changes at genomic markers between generations are not linked to changes in the frequencies at unknown QTL. That means that the metafounders derived by population–structure analysis genotypically exhibit no differences in trait means and no associated effects on phenotypic variance. In real life such a situation may possibly occur as a consequence of a trait not being under selection, a low trait heritability (not assumed here), or in a trait that is determined by few QTL only. However, for different origins, the mean trait level may differ even in the absence of a genetic trend in real breeding populations. Such a scenario was not specifically addressed by the way this simulation was implemented. Results shown therefore do not allow us to derive conclusions for such a scenario.

Macedo et al. (2022) reported different success rates with modelling of metafounders depending on whether a trait had a strong trend or a weak one. For traits with a high heritability (0.2–0.4), the use of metafounders improved slope estimates and reduced bias in other studies (Himmelbauer et al. 2024; van Grevenhof et al. 2019; Kudinov et al. 2022).

### 4.4 Aspects of Rescaling the Additive Genetic Variance

It is important to keep in mind that the rescaling used in this investigation is based on estimated elements of Γ, which are results of genomic relationships only and it is therefore invariant to whether the trait considered exhibits a genetic trend or not. With respect to the scaling of the additive‐genetic variance, the effect of modelling metafounders can be divided into two conceptual steps. With nMF = 1, there is an implicit (and indispensable) step of rescaling the additive‐genetic variance to the new base of G (a conceptual base population with 0.5 allele frequencies), leading to the initial ‘jump’ upwards for $\sigma_{a_{\text{resc}}}^{2}$ from 1 to 1.481 observed for nMF = 1, accompanied by an increase in heritability from 0.25 to 0.33 (assuming a stable residual variance). The introduction of structural information then leads to a continuous decrease from nMF = 1 onwards. This observation is in line with quantitative‐genetic theory describing the genetic variance in subdivided populations as the sum of within‐line variance and a between‐line component, the variance of line‐means (Falconer and Mackay 2009) (for the interested reader we added a more detailed discussion of this aspect as Appendix B). In this investigation we used the variance of TBV of PF as the initial parameter for the additive‐genetic variance to be used in the estimation of EBV. With this short‐cut we tried to mimic a situation where the parameter itself is estimated (without error) from a model assuming an unrelated and unstructured pedigree base. However, if this assumption does not hold, i.e., if the pedigree base is already structured, the variance of the line means adds to the estimate for the additive‐genetic variance. Recovering structural information and introducing it by changing the initially assumed covariance from A to $A^{\Gamma }$, modelling the variance of metafounder means becomes an inherent part of the coefficient matrix and its contribution to the parameter estimate has to be removed. This task is achieved by using the proposed rescaling of the initial parameter estimate (Legarra et al. 2015).

Discussion on the aspect of rescaling the additive‐genetic variance is scarce in the metafounder literature. Some authors explicitly mention the rescaling, while others do not, possibly because it is silently done by the software programs used (Himmelbauer et al. 2024). Kudinov et al. (2022) argue that only a homogeneous mixture of the base population in the current population would require the rescaling. Himmelbauer et al. (2024) recommended avoiding the rescaling altogether because it led to an overestimation of heritability in their simulation study. However, these authors and others (van Grevenhof et al. 2019) working with simulation studies reported true estimates of the additive‐genetic variance close to the ones calculated by the rescaling proposed by Legarra et al. (2015). Considering the argument above and the discussion in Appendix B, the apparent ‘overestimation’ in heritability might be the consequence of the initial step of rescaling to a base of 0.5 allele frequencies.

### 4.5 Validation Considering the True Genotypes of PF

Repeating the optimisation process with $\Gamma_{\text{true}}$, we observed only a slight improvement in prediction quality compared to estimated Γ and an overall very similar behaviour when using the same cluster definitions. This confirms the argument that metafounders are not evenly related to the genotyped population and single estimates of diagonal elements of Γ outside the parameter space do not necessarily have a great impact on the overall prediction quality of EBV. In any case, keeping only a short distance from genotyped population to PF seems to be an advisable strategy to avoid an undue accumulation of estimation errors. Other authors already reported positive effects of short pedigrees when implementing metafounders (Kudinov et al. 2022; Macedo et al. 2022).

### 4.6 Outlook: Application to Real Data

The proposed strategy of clustering imputed GT of PF in order to define metafounders and to assign PF to metafounders worked well in the study population, although it required considerable computing resources. For an application to real data, the number of PF vs. the available computing resources will be the limiting factor in most modern populations under selection. An investigation of different, more sophisticated clustering approaches dealing with large data was beyond the scope of this study. Several options might be considered: stepwise clustering, preliminary selection of representative animals, or a combination of different methods. A regression of genotypes on metafounder allele frequencies including an intercept in the model can give an indication as to whether all relevant stratifications in the genotyped population are covered by the metafounders defined so far (Chiang et al. 2010; He et al. 2018; Anglhuber et al. 2024). A large intercept for a group of animals would then suggest a stratification not yet considered. This might also be an approach to check if a re‐definition of metafounders is necessary when genotyped animals from outside the ‘core’ population are added to the pool, e.g., in a situation with across‐country breeding value estimation.

To avoid an accumulation of estimation errors, we recommend keeping as low a distance as possible from the genotyped population to the PF. Our approach here could be a beneficial general strategy, with a maximum of two generations between genotyped animals and the PF as a general guideline. However, in most (dairy) populations this will lead to a loss of pedigree and phenotypic data. Although in most populations also AI bulls born before the switch to genomic selection are genotyped, genotyping of females has only become more common in recent years. The consequences and strategies of a reduction in data have been investigated by other authors (i.e., Macedo et al. 2022; Lourenco et al. 2014).

An aspect more technical in nature is related to the use of G with 0.5 allele frequencies. Such a G is most likely singular and therefore not invertible. This might require the consideration of other models, replacing the standard Single‐Step (animal‐) model by alternatives like Single‐Step SNPBLUP (Fernando et al. 2014; Liu et al. 2014).

In a first moment, the method presented here may sound too complex for practical use, but first applications to real data from Brown Swiss cattle indicate that the approach can be implemented successfully with some adaptations to routine pipelines (ongoing research).

## 5 Conclusion

To answer the three questions raised in the introduction: 1. It is possible to define metafounders based on population structure analysis of the imputed genotypes of pedigree founders. Care needs to be taken to rescale the additive‐genetic variance, to correctly account for a structured base population in a model with metafounders. 2. Inbreeding coefficients and elements of Γ can be used to find an optimum nMF. 3. In a trait without genetic trend, prediction quality slightly deteriorated with the modelling of metafounders but was still stable in the range of the optimum nMF modelled. In a trait with a strong genetic trend, the modelling of many metafounders improved prediction quality compared to a common Single‐Step model. The largest improvement was achieved in the range of the proposed optimum nMF.

## Funding

This work was supported by Arbeitsgemeinschaft Süddeutscher Rinderzucht‐ und Besamungsorganisationen e.V.

## Conflicts of Interest

The authors declare no conflicts of interest.

## Figures and Tables

**FIGURE 13:**
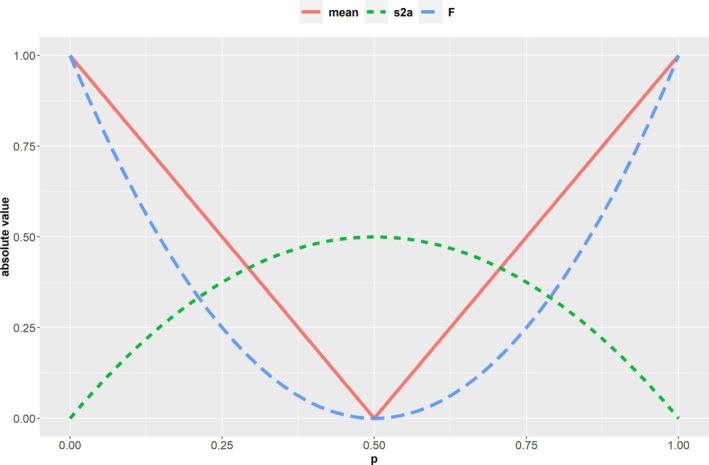
Effect on mean, variance and inbreeding when allele frequencies change from 0.5 towards fixation, effect of allele substitution *a* = 1. Mean is plotted as $\left|M\right|$. [Colour figure can be viewed at wileyonlinelibrary.com]

## Data Availability

Genotypes are property of the breeding organisations and therefore not publicly available.

## Appendix A

### Simulation of Genetic Trend

The following theoretical background for our proposal to simulate a trait with a (strong) trend is taken from Chapter 7 of Introduction to Quantitative Genetics (Falconer and Mackay 2009).

Assume the genotypic value of a favourable allele is a=1. The effect of the allele substitution α is dependent on the gene‐effect a, the dominance d and the allele frequencies p and q: $\alpha =a+d\left(q-p\right)$. The contribution of the gene to the population mean is defined as $M=a\left(p-q\right)+2\mathrm{dpq}$. The additive genetic variance at a locus is $\sigma_{a}^{2}=2\mathrm{pq\alpha}2$. In the absence of dominance effects which is assumed here d=0, α=a and $M=a\left(p-q\right)$.

In a hypothetical base‐population with p=q=0.5 the contribution of the gene to the additive genetic variance of the trait $\sigma_{a}^{2}$ is at maximum and there is no contribution of the gene to the mean trait‐value of the population (M=0). With the departure of allele frequencies from 0.5 towards fixation (either 0 or 1), the contribution of the gene to $\sigma_{a}^{2}$ decreases, while at the same time the contribution to the population mean M grows linearly (Figure 13).

In classic quantitative genetics theory, inbreeding does not directly relate to allele‐frequency changes but is defined in terms of deviation from Hardy–Weinberg‐Equilibrium, i.e., an excess of homozygotes (Falconer and Mackay 2009). In this context inbreeding can only affect M, if d≠0.

However, genomic inbreeding coefficients do consistently reflect allele‐frequency changes and corresponding effects on genotype frequencies even under HW assumptions. This is because the arising of inbreeding (F) is calculated as F=1−2pq/0.5, in relation to a base with allele frequencies of 0.5. Therefore, any systematic allele frequency change towards the fixation of either allele leads to a departure from maximum heterozygosity and increases genomic F. This increase follows a parabola form (Figure 13).

In consequence, only loci with a consistent change in allele frequencies from a medium value towards fixation can have a consistent effect on the mean value of the locus in the population (‘genetic trend’).

To detect SNPs to be defined as QTL for a trait showing a consistent trend, we first had to identify SNP with a consistent allele‐frequency change over time. We regressed gene‐contents at each SNP on a vector of birth year classes (2012 to 2024) in our data. A continuous change in allele‐frequencies was assumed when the fit of this regression (*R* ^2^) was at least 0.3 simultaneously excluding SNPs whose allele frequencies crossed the 0.5 maximum, e.g., from p=0.4 to p=0.6. In this way we identified 22,964 SNPs capable of producing a consistent genetic trend and selected 1000 at random once and checked the distribution among the chromosomes. LD between markers was not considered in this sampling scheme. Gene‐effects for these QTL‐SNPs were sampled from $N\left(0,\sqrt{\left(2*\sigma_{a}^{2}/1,000\right)}\;\right)$, with $\sigma_{a}^{2}$ = 1. This was repeated 500 times for each animal. The sign of the gene‐effect was then aligned to the direction of allele‐frequency change (increase in frequency of the reference‐allele resulted in positive gene‐effect, a decrease in a negative). TBV were then calculated as the product of gene‐effect times gene‐content at that locus and summed up. Finally mean TBV of PF were set to 0 and all TBV were rescaled by the variance observed in the TBV of PF. Phenotypes were calculated as TBV + random factor drawn from $N\left(0,\sqrt{\sigma_{e}^{2}}\right)$, with $\sigma_{e}^{2}$ set to fulfil: $h^{2}=\frac{\sigma_{a}^{2}}{\sigma_{a}^{2}+\sigma_{e}^{2}}=0.25$ in PF. This simulation resulted in a trend corresponding to 0.2 standard deviation per year (Figure 7).

## Appendix B

### Rescaling of the Additive Genetic Variance

As we pointed out in our previous publication (Anglhuber et al. 2024) the Γ matrix is commonly calculated referencing to a purely conceptual remote base population with maximum heterozygosity (under Hardy–Weinberg conditions) of 0.5 ($H_{0.5}$). This allows to use the assumption of a uniform ‘base’‐allele‐frequency of 0.5 when setting up matrix G, which in turn assures that the inbreeding coefficients provided by G are meaningful measures of individual homozygosity. Applying Γ to calculate $A^{\Gamma }$ therefor involves two mutual independent steps of transformation, where the first one is to scale the numerator relationship matrix A, that was build referencing to an existing pedigree base (that in most cases will have a true heterozygosity $H_{\mathrm{PB}}<H_{0.5}$) to this new conceptual base population with $H_{0.5}$ (Anglhuber et al. 2024). The second step of transformation is to introduce information about stratification (here termed ‘structural information’) into A, which basically means that pedigree founders (that were assumed to be unrelated, with zero inbreeding and in HWE by the standard‐model) are now related or ‘structured’ to some degree. (Or, to put that more formally for a single locus: the mean of all heterozygosities across strata does not equal the expected heterozygosity based on the mean allele frequency in the base‐population, in simplified notation $2\bar{p}\bar{q}\neq \bar{2\mathrm{pq}}$).

Both steps of transformation are also inherently applied when scaling the additive‐genetic variance parameter used by the model to accommodate the new assumptions about the pedigree base. The final rescaled parameter then virtually answers the question how to adopt the additive‐genetic variance, estimated from a structured base‐population with $H_{\mathrm{PB}}<H_{0.5}$ to a new conceptual base‐population that is unstructured and has $H_{0.5}$. Again, both steps of transformation are mutually independent and can be applied separately. This separation is useful to elucidate aspects that are not obvious otherwise.

Legarra et al. (2015) gave an elaborated derivation for the rescaling of the additive‐genetic variance. He proposed to scale the additive genetic variance based on the estimated Γ matrix in the following way:

$$\sigma_{a_{\text{resc}}}^{2}=\frac{\sigma_{a}^{2}}{\left(1-\frac{\bar{\mathrm{diag}\left(\Gamma \right)}}{2}+\bar{\Gamma }\right)}$$

It is not immediately obvious how this formulation relates to text‐book versions that describe the evolution of the additive genetic variance of a large random mating base‐population when it divides into several inbred strains or lines. To discuss the subject further it might be helpful to first change the above formulation to give it more ‘text‐book‐like’ appearance

$$\sigma_{a}^{2}=\left(1-\bar{F\;}+2\bar{f}\;\right)\sigma_{a_{\text{resc}}}^{2}$$

This is because diagonals of Γ are self‐relationships of metafounders equaling to two times the inbreeding coefficient and off‐diagonals represent relationships between metafounders which equals to two times the coancestry between metafounders (f). With increasing nMF the mean of Γ becomes more and more dominated by the mean of the off‐diagonals and so $\bar{\Gamma }\to 2\bar{f}$. Omitting the ‘average’ notation bar for convenience, text‐book formulations in its simplest form give $\left(2F\right)\sigma_{a_{\text{resc}}}^{2}$ for the between‐lines component, $\left(1-F\right)\sigma_{a_{\text{resc}}}^{2}$ for the within‐lines component and $\left(1+F\right)\sigma_{a_{\text{resc}}}^{2}$ for the total variance (e.g., (Falconer and Mackay 2009), p. 265). As an illustration we applied both approaches on stored estimated Γ matrices for nMF = 1 to 1400 from our optimisation run (example with genetic trend) and compared Legarra et al.'s (2015) formulation to text‐book formulations for within‐lines component (for nMF = 1) and total variance (for nMF > 1). Results of this comparison for the rescaled variance and rescaled heritability based hereon ($h_{\text{resc}}^{2}$) are displayed in Table 9 columns 2 to 5. When applying text‐book formulations we observe a severe drop in rescaled variance for nMF > 1, much stronger than observed for Legarra et al.'s (2015) formulation that provide a more continuous decrease in rescaled variance. Obviously both formulations perform differently when compared in this manner. This is, however, misleading.

**TABLE 9:** Results for rescaled variances and heritabilities when based on calculation of Γ matrices that include an additional transformation step to a conceptual base‐population with maximum heterozygosity under HWE (columns 2 to 5) or when this additional rescaling step is omitted (columns 6 to 9). The parameter for additive‐genetic variance estimated from the pedigree base is set to one. Residual variance is adjusted to provide a trait heritability of 0.25 in the pedigree base.

nMF	Reference to $H_{0.5}$				Reference to $H_{\mathrm{PB}}$			
	Legarra		Textbook		Legarra		Textbook	
	$\sigma_{a_{\text{resc}}}^{2}$	$h_{\text{resc}}^{2}$	$\sigma_{a_{\text{resc}}}^{2}$	$h_{\text{resc}}^{2}$	$\sigma_{a_{\text{resc}}}^{2}$	$h_{\text{resc}}^{2}$	$\sigma_{a_{\text{resc}}}^{2}$	$h_{\text{resc}}^{2}$
1	1.481	0.331	1.481	0.331	1.000	0.250	1.000	0.250
200	1.107	0.270	0.644	0.177	0.748	0.199	0.747	0.199
400	1.002	0.250	0.607	0.168	0.677	0.184	0.677	0.184
600	0.950	0.240	0.587	0.164	0.641	0.176	0.641	0.176
800	0.904	0.232	0.570	0.160	0.611	0.169	0.611	0.169
1000	0.859	0.223	0.551	0.155	0.580	0.162	0.581	0.162
1200	0.804	0.211	0.529	0.150	0.543	0.153	0.544	0.154
1400	0.683	0.185	0.474	0.136	0.461	0.133	0.462	0.134

In a second approach, we applied only the introduction of structural information into A without performing the additional step of rescaling to $H_{0.5}$, that means we referenced Γ to the expected heteroygosity based on estimated allele‐frequencies of the pedigree base $E\left(H_{\mathrm{PB}}\right)=2\bar{p}\bar{q}=0.338$. In this case we observe both approaches to provide identical results (Table 9 columns 6 to 9).

From these observations we derive the following conclusions: First, Legarra et al.'s (2015) approach (which virtually balances diagonals and off‐diagonals of Γ) provide consistent results irrespective of whether global rescaling is performed simultaneously with the introduction of stratification information or not (please note that column 6 equals column 2 when divided by 1.481) whereas simplifying text‐book formulations based on diagonals alone perform correctly only without rescaling to $H_{0.5}$. With respect to the isolated introduction of structural information (which, after all, is the fundamental purpose of metafounder) and the corresponding effect on the additive genetic variance both approaches therefor provide identical results. Second, since global rescaling to $H_{0.5}$ is mostly artificial in nature and is only needed to adopt $A^{\Gamma }$ to a G matrix calculated using the assumption of a uniform ‘base’‐allele‐frequency of 0.5, it is a dispensable step. Then, the presence of a structured pedigree base per se will always lead to a rescaled variance that is *smaller* than the parameter estimate derived from the structured base‐population. This is a consequence of diverging fixation processes between lines and the additional variation of line‐means that will contribute to the estimate derived from the pedigree base. Since these components of variance are not present in a homogeneous, random‐mating base‐population with the same average allele‐frequencies, the associated parameter has to be smaller. This conclusion is in contrast to explanations given by Legarra et al. (2015).

## References

[jbg70039-bib-0001] Aldridge, M. N. , J. Vandenplas , and M. P. L. Calus . 2019. “Efficient and Accurate Computation of Base Generation Allele Frequencies.” Journal of Dairy Science 102, no. 2: 1364–1373. 10.3168/jds.2018-15264. 30471906

[jbg70039-bib-0002] Anglhuber, C. , C. Edel , E. C. G. Pimentel , R. Emmerling , K.‐U. Götz , and G. Thaller . 2024. “Definition of Metafounders Based on Population Structure Analysis.” Genetics Selection Evolution 56, no. 1: 43. 10.1186/s12711-024-00913-7. PMC11536677 38844876

[jbg70039-bib-0003] Bermann, M. , D. Lourenco , V. Breen , R. Hawken , F. Brito Lopes , and I. Misztal . 2021. “Modeling Genetic Differences of Combined Broiler Chicken Populations in Single‐Step GBLUP.” Journal of Animal Science 99, no. 4: skab056. 10.1093/jas/skab056. 33649764 PMC8355479

[jbg70039-bib-0004] Bradford, H. L. , Y. Masuda , P. M. VanRaden , A. Legarra , and I. Misztal . 2019. “Modeling Missing Pedigree in Single‐Step Genomic BLUP.” Journal of Dairy Science 102, no. 3: 2336–2346. 10.3168/jds.2018-15434. 30638995

[jbg70039-bib-0005] Chiang, C. W. K. , Z. K. Z. Gajdos , J. M. Korn , et al. 2010. “Rapid Assessment of Genetic Ancestry in Populations of Unknown Origin by Genome‐Wide Genotyping of Pooled Samples.” PLoS Genetics 6, no. 3: e1000866. 10.1371/journal.pgen.1000866. 20221249 PMC2832667

[jbg70039-bib-0006] Christensen, O. F. 2012. “Compatibility of Pedigree‐Based and Marker‐Based Relationship Matrices for Single‐Step Genetic Evaluation.” Genetics Selection Evolution 44, no. 1: 37. 10.1186/1297-9686-44-37. PMC3549765 23206367

[jbg70039-bib-0007] Christensen, O. F. , and M. S. Lund . 2010. “Genomic Prediction When Some Animals Are Not Genotyped.” Genetics Selection Evolution 42, no. 1: 2. 10.1186/1297-9686-42-2. PMC2834608 20105297

[jbg70039-bib-0008] Edel, C. , E. C. G. Pimentel , L. Plieschke , R. Emmerling , and K.‐U. Götz . 2016. “Short Communication: The Effect of Genotyping Cows to Improve the Reliability of Genomic Predictions for Selection Candidates.” Journal of Dairy Science 99, no. 3: 1999–2004. 10.3168/jds.2015-10246. 26723131

[jbg70039-bib-0009] Falconer, D. S. , and T. Mackay . 2009. Introduction to Quantitative Genetics. 4th ed. Harlow. 10.1093/genetics/167.4.1529 PMC1471025 15342495

[jbg70039-bib-0010] Fernando, R. L. , J. C. Dekkers , and D. J. Garrick . 2014. “A Class of Bayesian Methods to Combine Large Numbers of Genotyped and Non‐Genotyped Animals for Whole‐Genome Analyses.” Genetics Selection Evolution 46, no. 1: 50. 10.1186/1297-9686-46-50. PMC4262255 25253441

[jbg70039-bib-0011] Forni, S. , I. Aguilar , and I. Misztal . 2011. “Different Genomic Relationship Matrices for Single‐Step Analysis Using Phenotypic, Pedigree and Genomic Information.” Genetics Selection Evolution 43, no. 1: 1. 10.1186/1297-9686-43-1. PMC3022661 21208445

[jbg70039-bib-0012] Garcia‐Baccino, C. A. , A. Legarra , O. F. Christensen , et al. 2017. “Metafounders Are Related to F St Fixation Indices and Reduce Bias in Single‐Step Genomic Evaluations.” Genetics Selection Evolution 49, no. 1: 34. 10.1186/s12711-017-0309-2. PMC5439149 28283016

[jbg70039-bib-0013] Gengler, N. , P. Mayeres , and M. Szydlowski . 2007. “A Simple Method to Approximate Gene Content in Large Pedigree Populations: Application to the Myostatin Gene in Dual‐Purpose Belgian Blue Cattle.” Animal 1, no. 1: 21–28. 10.1017/S1751731107392628. 22444206

[jbg70039-bib-0014] van Grevenhof, E. M. , J. Vandenplas , and M. P. L. Calus . 2019. “Genomic Prediction for Crossbred Performance Using Metafounders.” Journal of Animal Science 97, no. 2: 548–558. 10.1093/jas/sky433. 30423111 PMC6358227

[jbg70039-bib-0015] Hartigan, J. A. , and M. A. Wong . 1979. “Algorithm AS 136: A K‐Means Clustering Algorithm.” Applied Statistics 28, no. 1: 100. 10.2307/2346830.

[jbg70039-bib-0016] He, J. , Y. Guo , J. Xu , et al. 2018. “Comparing SNP Panels and Statistical Methods for Estimating Genomic Breed Composition of Individual Animals in Ten Cattle Breeds.” BMC Genetics 19, no. 1: 56. 10.1186/s12863-018-0654-3. 30092776 PMC6085684

[jbg70039-bib-0017] Himmelbauer, J. , H. Schwarzenbacher , C. Fuerst , and B. Fuerst‐Waltl . 2024. “Exploring Unknown Parent Groups and Metafounders in Single‐Step Genomic BLUP: Insights From a Simulated Cattle Population.” Journal of Dairy Science 107, no. 10: 8170–8192. 10.3168/jds.2024-24891. 38908687

[jbg70039-bib-0018] Kudinov, A. A. , M. Koivula , G. P. Aamand , I. Strandén , and E. A. Mäntysaari . 2022. “Single‐Step Genomic BLUP With Many Metafounders.” Frontiers in Genetics 13: 1012205. 10.3389/fgene.2022.1012205. 36479243 PMC9721289

[jbg70039-bib-0019] Kudinov, A. A. , E. A. Mäntysaari , G. P. Aamand , P. Uimari , and I. Strandén . 2020. “Metafounder Approach for Single‐Step Genomic Evaluations of Red Dairy Cattle.” Journal of Dairy Science 103, no. 7: 6299–6310. 10.3168/jds.2019-17483. 32418688

[jbg70039-bib-0020] Legarra, A. , I. Aguilar , and I. Misztal . 2009. “A Relationship Matrix Including Full Pedigree and Genomic Information.” Journal of Dairy Science 92, no. 9: 4656–4663. 10.3168/jds.2009-2061. 19700729

[jbg70039-bib-0021] Legarra, A. , M. Bermann , Q. Mei , and O. F. Christensen . 2024a. “Estimating Genomic Relationships of Metafounders Across and Within Breeds Using Maximum Likelihood, Pseudo‐Expectation–Maximization Maximum Likelihood and Increase of Relationships.” Genetics Selection Evolution 56, no. 1: 35. 10.1186/s12711-024-00892-9. PMC11536831 38698347

[jbg70039-bib-0022] Legarra, A. , M. Bermann , Q. Mei , and O. F. Christensen . 2024b. “Redefining and Interpreting Genomic Relationships of Metafounders.” Genetics, Selection, Evolution GSE 56, no. 1: 34. 10.1186/s12711-024-00891-w. 38698373 PMC11536960

[jbg70039-bib-0023] Legarra, A. , O. F. Christensen , Z. G. Vitezica , I. Aguilar , and I. Misztal . 2015. “Ancestral Relationships Using Metafounders: Finite Ancestral Populations and Across Population Relationships.” Genetics 200, no. 2: 455–468. 10.1534/genetics.115.177014. 25873631 PMC4492372

[jbg70039-bib-0024] Liu, Z. , M. E. Goddard , F. Reinhardt , and R. Reents . 2014. “A Single‐Step Genomic Model With Direct Estimation of Marker Effects.” Journal of Dairy Science 97, no. 9: 5833–5850. 10.3168/jds.2014-7924. 25022678

[jbg70039-bib-0025] Lourenco, D. A. L. , I. Misztal , S. Tsuruta , et al. 2014. “Are Evaluations on Young Genotyped Animals Benefiting From the Past Generations?” Journal of Dairy Science 97, no. 6: 3930–3942. 10.3168/jds.2013-7769. 24679931

[jbg70039-bib-0026] Lozada‐Soto, E. A. , F. Tiezzi , J. Jiang , J. B. Cole , P. M. VanRaden , and C. Maltecca . 2022. “Genomic Characterization of Autozygosity and Recent Inbreeding Trends in All Major Breeds of US Dairy Cattle.” Journal of Dairy Science 105, no. 11: 8956–8971. 10.3168/jds.2022-22116. 36153159

[jbg70039-bib-0027] Macedo, F. L. , J. M. Astruc , T. H. E. Meuwissen , and A. Legarra . 2022. “Removing Data and Using Metafounders Alleviates Biases for All Traits in Lacaune Dairy Sheep Predictions.” Journal of Dairy Science 105, no. 3: 2439–2452. 10.3168/jds.2021-20860. 35033343

[jbg70039-bib-0028] Mäntysaari, E. A. , Z. Liu , and P. M. VanRaden . 2010. “Interbull Validation Test for Genomic Evaluations.” Interbull Bulletin 41: 17–22.

[jbg70039-bib-0029] Meuwissen, T. H. E. , T. Luan , and J. A. Woolliams . 2011. “The Unified Approach to the Use of Genomic and Pedigree Information in Genomic Evaluations Revisited.” Journal of Animal Breeding and Genetics 128, no. 6: 429–439. 10.1111/j.1439-0388.2011.00966.x. 22059576

[jbg70039-bib-0030] Morissette, L. , and S. Chartier . 2013. “The k‐Means Clustering Technique: General Considerations and Implementation in Mathematica.” TQMP 9, no. 1: 15–24. 10.20982/tqmp.09.1.p015.

[jbg70039-bib-0031] Pérez‐Enciso, M. 1995. “Use of the Uncertain Relationship Matrix to Compute Effective Population Size.” Journal of Animal Breeding and Genetics 112, no. 1–6: 327–332. 10.1111/j.1439-0388.1995.tb00574.x.

[jbg70039-bib-0032] Plieschke, L. , C. Edel , E. C. G. Pimentel , R. Emmerling , J. Bennewitz , and K.‐U. Götz . 2016. “Systematic Genotyping of Groups of Cows to Improve Genomic Estimated Breeding Values of Selection Candidates.” Genetics Selection Evolution 48, no. 1: 73. 10.1186/s12711-016-0250-9. PMC5039940 27677439

[jbg70039-bib-0033] Quaas, R. L. , and E. J. Pollak . 1981. “Modified Equations for Sire Models With Groups.” Journal of Dairy Science 64, no. 9: 1868–1872. 10.3168/jds.S0022-0302(81)82778-6.

[jbg70039-bib-0034] R Core Team . 2018. R: A Language and Environment for Statistical Computing. R Foundation for Statistical Computing.

[jbg70039-bib-0035] Teegen, R. , C. Edel , and G. Thaller . 2009. “Population Structure of the Trakehner Horse Breed.” Animal 3, no. 1: 6–15. 10.1017/S1751731108003273. 22444167

[jbg70039-bib-0036] VanRaden, P. M. 2008. “Efficient Methods to Compute Genomic Predictions.” Journal of Dairy Science 91, no. 11: 4414–4423. 10.3168/jds.2007-0980. 18946147

[jbg70039-bib-0037] Vitezica, Z. G. , I. Aguilar , I. Misztal , and A. Legarra . 2011. “Bias in Genomic Predictions for Populations Under Selection.” Genetics Research 93, no. 5: 357–366. 10.1017/S001667231100022X. 21767459

[jbg70039-bib-0038] Wickham, H. 2016. ggplot2: Elegant Graphics for Data Analysis. 2nd ed. Springer‐Verlag.

